# Expanding the archaellum regulatory network – the eukaryotic protein kinases ArnC and ArnD influence motility of *Sulfolobus acidocaldarius*

**DOI:** 10.1002/mbo3.414

**Published:** 2016-10-22

**Authors:** Lena Hoffmann, Andreas Schummer, Julia Reimann, Maria F. Haurat, Amanda J. Wilson, Morgan Beeby, Bettina Warscheid, Sonja‐V. Albers

**Affiliations:** ^1^ Molecular Biology of ArchaeaInstitute of Biology IIFaculty of BiologyMicrobiologyUniversity of FreiburgFreiburgGermany; ^2^ Department of Biochemistry and Functional ProteomicsInstitute of Biology IIFaculty of BiologyUniversity of FreiburgFreiburgGermany; ^3^ Department of Life SciencesImperial College of LondonLondonUK; ^4^ BIOSS Centre for Biological Signaling StudiesUniversity of FreiburgFreiburgGermany

**Keywords:** archaeal flagella, archaellum regulation, phosphorylation, protein kinases, *S. acidocaldarius*, signaling network

## Abstract

Expression of the archaellum, the archaeal‐type IV pilus‐like rotating motility structure is upregulated under nutrient limitation. This is controlled by a network of regulators, called the archaellum regulatory network (arn). Several of the components of this network in *Sulfolobus acidocaldarius* can be phosphorylated, and the deletion of the phosphatase PP2A results in strongly increased motility during starvation, indicating a role for phosphorylation in the regulation of motility. Analysis of the motility of different protein kinase deletion strains revealed that deletion of *saci_0965*,* saci_1181,* and *saci_1193* resulted in reduced motility, whereas the deletion of *saci_1694* resulted in hypermotility. Here ArnC (Saci_1193) and ArnD (Saci_1694) are characterized. Purified ArnC and ArnD phosphorylate serine and threonine residues in the C‐terminus of the repressor ArnB. *arnC* is upregulated in starvation medium, whereas arnD is constitutively expressed. However, while differences in the expression and levels of *flaB* were observed in the *ΔarnD* strain during growth under rich conditions, under nutrient limiting conditions the *ΔarnC* and *ΔarnD* strains showed no large differences in the expression levels of the archaellum or of the studied regulators. This suggests that next to the regulation via the archaellum regulatory network additional regulatory mechanisms of expression and/or activity of the archaellum exist.

## 1 Introduction

Signal transduction and integration is a crucial process for all organisms which, for example, allows them to react to changing environmental conditions. In general, environmental signals are received by membrane‐bound receptors, which transduce the signal to receiver proteins that alter gene expression, which ultimately leads to adaptation. In this process, posttranslational modifications, specifically phosphorylation and de‐phosphorylation are often employed as they can rapidly change the activity, function, and interactions of a protein in a reversible manner. Here, we study the regulation of the expression of components of the archaellum, the archaeal motility structure. The archaellum consists of seven proteins and is highly upregulated under starvation conditions. The genes encoding the archaellum proteins are organized in an operon. (Lassak et al., 2012). A recent study showed that phosphorylation of regulators involved in the expression of the components of the archaellum in *Sulfolobus acidocaldarius* might play an important role in this process (Reimann et al., 2012). In *Sulfolobales,* the currently identified kinases belong to the group of the eukaryotic or Hanks‐type protein kinases (ePKs) (Esser et al., 2011).

The extensive family of ePKS proteins is defined by a conserved catalytic core of approximately 250–300 amino acid residues which is found in both serine/threonine and tyrosine protein kinases. This kinase domain can occur alone or together with other functional domains, and the kinase can be present as monomer, dimer or associated with other proteins. A typical ePK domain folds into two lobes, a smaller N‐terminal and larger C‐terminal lobe (Hanks & Hunter, 1995). While the N‐terminal ß‐sheet‐rich lobe binds and orients ATP and the divalent cation, the C‐terminal lobe, that almost exclusively contains α‐helices, is involved in binding the peptide substrate and in phosphotransfer (Hanks & Hunter, 1995). The domain is further characterized by the presence of 12 subdomains. Subdomains I–IV are located in the N‐terminal lobe and include the glycine‐rich loop involved in orientation of the nucleotide as well as invariant lysine and aspartate residues necessary for ATP binding and stability. Subdomains V‐XII are located in the C‐terminal lobe and contribute mainly to either structural stability, ion chelation or formation of the catalytic and activation loops. ePKs are classified into different groups according to the residues located in subdomains VIb and VIII (Hanks, 2003; Hanks & Hunter, 1995; Johnson, Lowe, Noble, & Owen, 1998).

In eukaryotes, protein phosphorylation is almost exclusively found on Ser, Thr and to a lower extend on Tyr residues. Phosphorylation of these residues is mainly carried out by typical ePKs, however, also several protein kinase families that lack some of the subdomains of typical ePKs have been identified. These proteins are called atypical eukaryotic protein kinases (aPKs) (Laronde‐LeBlanc & Wlodawer, 2005a; LaRonde‐LeBlanc & Wlodawer, 2005b; Leonard, Aravind, & Koonin, 1998).

For a long time it was accepted that ePKs were exclusively present in eukaryotes, but it has become clear that they are also found in bacteria and archaea. While bacteria employ homologs of eukaryotic‐type Ser/Thr kinases (eSTKs), they evolved a different type of tyrosine kinase (called BY‐kinase) (Grangeasse, Nessler, & Mijakovic, 2012; Pereira, Goss, & Dworkin, 2011). BY kinases and eSTKs are involved in various processes like virulence, antibiotic‐resistance and production as well as in the regulation of DNA‐binding of transcription factors (Kobir et al., 2011; Dworkin, 2015; Kalantari et al., 2015). However, most bacterial signal transduction cascades rely on classical two component systems. Interestingly, eSTKs and BY kinases can integrate with two component systems to create additional layers of regulation, for example, by phosphorylating response regulators on additional Ser, Thr or Tyr residues (Burnside & Rajagopal, 2012).

Protein phosphorylation and signaling cascades in archaea have not been studied in as much detail as in eukaryotes and bacteria. In archaea, the chemotaxis system in *Halobacterium salinarum* is probably the best studied example. It is very similar to chemotaxis systems in bacteria like *Escherichia coli*, including the central CheA‐CheY two component system (Rudolph & Oesterhelt, 1996; Rudolph, Tolliday, Schmitt, Schuster, & Oesterhelt, 1995; Schlesner et al., 2012). In contrast to euryarchaea, which seem to have acquired two component systems by horizontal gene transfer, the second large archaeal phylum, the crenarchaeota lack two component systems (Ashby, 2006; Koretke, Lupas, Warren, Rosenberg, & Brown, 2000; Ponting, Aravind, Schultz, Bork, & Koonin, 1999).

Interestingly, ePKs and aPKs are present in the genomes of many archaea (Esser et al., 2016; Kennelly, 2014). Our knowledge of crenarchaeal ePKs derives mainly from studies performed on kinases of *S. solfataricus*. Several ePKs and an aPK of *S. solfataricus* have been characterized with respect to their substrate specificity, ion preference as well as autophosphorylation and phosphotransfer activities to various exogenous substrates as, for example, casein (Haile & Kennelly, 2011; Lower, Bischoff, & Kennelly, 2000; Lower & Kennelly, 2002, 2003; Lower, Potters, & Kennelly, 2004; Ray, Potters, Haile, & Kennelly, 2015). However, little is known about which proteins are the natural targets of these kinases. St0829 of *Sulfolobus tokodaii* was demonstrated to be a Ser/Thr‐specific kinase that phosphorylates the forkhead‐associated (FHA) domain‐containing protein St1565. Unphosphorylated St1565 binds to the promoter region of the operon encoding the archaellum, while phosphorylation inhibits binding (Duan & He, 2011; Wang, Yang, Zhang, & He, 2010). However, the physiological function of this kinase remains unclear.

**Figure 1:**
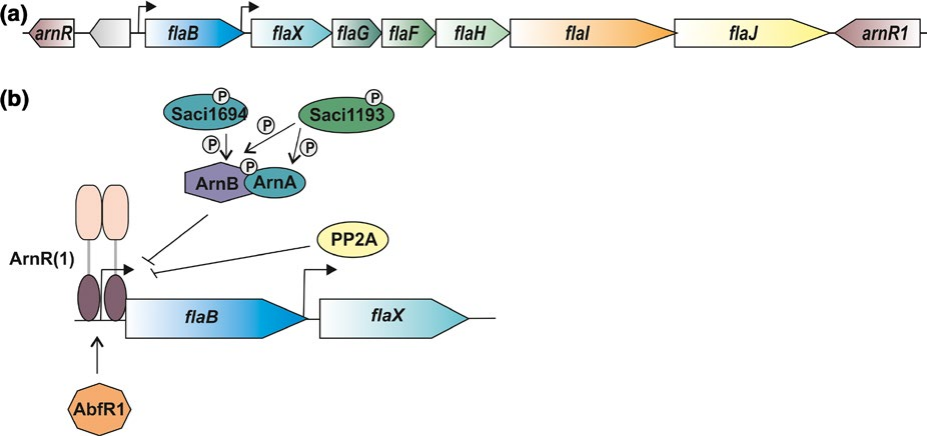
Archaellum operon and archaellum regulatory network. (a) The operon from *flaB* to *flaJ* encodes the subunits of the archaellum of *S. acidocaldarius*. Upstream and downstream of the operon, the activators *arnR* and *arnR1* are encoded. (b) Model of the archaellum regulatory network (arn) and the proteins involved in archaellum expression during starvation. The repressors ArnA and ArnB are phosphorylated by ePKs and interact to block expression if nutrients are present. The phosphatase Saci_PP2A also represses expression of the archaellin FlaB. During starvation, the positive regulators ArnR and ArnR1 activate expression by binding to the *flaB* promoter. Additionally, the biofilm regulator AbfR1 contributes to stimulation of archaellum expression (adapted from Albers & Jarrell, 2015)

An analysis of the genome of *S. acidocaldarius* identified several possible Ser/Thr and Tyr kinases and two phosphatases (Esser et al., 2012). Most of the identified protein kinases belong to the typical ePKs of the Hanks‐type and, based on sequence homology and conservation of specific motifs, two of them belong to the aPKs of the RIO type (Rio 1 and Rio 2) (Esser & Siebers, 2013). The two phosphatases, Saci_PP2A and Saci_PTP, were characterized as a Ser/Thr‐specific and a dual‐specific phosphatase, respectively (Reimann et al., 2013). Remarkably, a study of the phosphoproteome of *S. acidocaldarius* showed that half of the identified phosphopeptides were phosphorylated on tyrosine residues, but, whereas the deletion of *saci_pp2a* had a major impact on growth and the appearance of the cells, no effects of deletion of *saci_ptp* were observed (Reimann et al., 2013). Currently, the only protein kinases of *S. acidocaldarius* that have been studied in some detail are Saci_1193 and Saci_1694. These studies showed that Saci_1193 phosphorylates the repressors of archaellum expression, ArnA and ArnB, whereas Saci_1694 phosphorylates ArnB, but not ArnA (Reimann et al., 2012). The archaellum, the motility structure of *S. acidocaldarius,* is encoded by the archaellum operon which contains seven genes (*flaB, X, G, F, H, I,* and *J*), that are transcribed by two promoters (Figure 1a, (Lassak et al., 2012)). The promoter upstream of the gene encoding FlaB, the filament protein of the archaellum, is strongly induced during starvation (Lassak, Peeters, Wróbel, & Albers, 2013). A network of positive and negative regulators is necessary for archaellum expression (Albers & Jarrell, 2015). ArnA and ArnB, which are encoded elsewhere in the genome, form a complex which represses expression of the archaellum (Figure 1b, Reimann et al., 2012). Furthermore, ArnR and ArnR1, which are encoded in the direct vicinity of the archaellum operon, are membrane‐bound transcription factors which induce the expression of the archaellum by binding to specific boxes within the promoter region of *flaB* (Lassak et al., 2013). Interestingly, ArnR1 can also be phosphorylated in vivo (Reimann et al., 2013). A major negative regulator of biofilm production, AbfR1, is also a positive regulator of the archaellum operon, and was shown to be phosphorylated *in vivo*, too (Orell et al., 2013). The importance of phosphorylation in the regulation of motility in *S. acidocaldarius* is further emphasized by the observation that deletion of *saci_pp2a* results in a strongly increased motility during starvation (Reimann et al., 2013).

Here, we aimed to generate deletion mutants of the protein kinases of *S. acidocaldarius* and analyzed the mutants obtained in motility assays. Two of the identified kinases, ArnC (Saci_1193) and ArnD (Saci_1694) were characterized with respect to their function in the regulation of the expression and function of the archaellum.

## 2 Experimental Procedures

### 2.1 Strains and growth conditions

Strains used in this study are described in Table S1. *S. acidocaldarius* markerless in‐frame deletion mutants and uracil auxotrophic strains were grown at 75°C in basal Brock medium (pH 3.5) supplemented with 0.1% NZ‐amine, 0.2% dextrin, and 10 μg/ml uracil (Brock, Brock, Belly, & Weiss, 1972; Wagner et al., 2012). Strains containing a plasmid were grown in the absence of uracil.

To compare cells under rich and starvation conditions, strains were grown in 250 ml Brock medium supplemented with 0.1% NZ‐amide and 0.2% dextrin to an OD_600_ of 0.4. Cells were centrifuged at 75°C for 10 min at 4.100 g using a Thermocentrifuge (Hettrich). Subsequently, the pellet was either resuspended in nutrient‐rich (0.1% NZ‐amine, 0.2% dextrin) or nutrient‐poor medium (basal brock medium), and growth was continued.

### 2.2 Construction of plasmids

Plasmids used in this study are described in Table S2. Cloning was performed in *E. coli* Top10. Plasmids used to construct deletion mutants (pSVA1019, pSVA1076, pSVA1036, pSVA1038, pSVA1080, pSVA1087, pSVA1088, pSVA1091, pSVA2287) were created by fusing the upstream and downstream regions of the genes of interest via overlap PCR and subsequent cloning of the resulting fragment into pSVA406. Plasmids used for complementation of the deletion mutants (pSVA3203 and pSVA3208) were constructed by inserting a PCR fragment containing the gene of interest with a C‐terminal HA‐tag and a ~300 bp upstream region into pSVA1551. pSVA1551 contains a maltose inducible promoter, a TEV protease cleavage site and a C‐terminal StrepII and His10 tag. During cloning all these parts were removed from the plasmid and the kinase gene with its own promoter and C‐terminal HA‐tag sequence was cloned into pSVA1551. The primers and restriction sites used are described in Table S3.

### 2.3 Construction of in‐frame deletion mutants of *S. acidocaldarius*

Plasmids were methylated using *E. coli* ER1821, and transformation of *S. acidocaldarius* with these plasmids was performed essentially as described (Wagner et al., 2012).

### 2.4 Motility assays

Motility assay were essentially performed as described previously (Lassak et al., 2012). For a detailed description see supporting information. Motility assays were performed with mutants in the MW001 background strain which has a reduced motility compared to its parental strain DSM639.

### 2.5 Expression and purification of ArnB, ArnC,and ArnD

All proteins were expressed in *E. coli* Rosetta pLysS strain using plasmids pSVA1036 (*arnB*), pSVA1009 (*arnC*), and pSVA1076 (*arnD*). Expression and purification was performed as described (Reimann et al., 2012).

### 2.6 *In vitro* phosphorylation

In vitro phosphorylation assays, were essentially performed as described before (Reimann et al., 2012). Briefly, 1 μg ArnB was mixed with either 20 ng or 2 ng ArnC in buffer containing 150 mmol/L KCl, 50 mmol/L HEPES pH 7.8, or with 20 ng or 2 ng ArnD in buffer containing 150 mmol/L KCl, 25 mmol/L MES pH 6.5. Samples were supplemented with 1 mmol/L MnCl_2_ and 1 mmol/L ATP and incubated for 30 min at 55°C.

### 2.7 Mass spectrometry

Proteins were acetone‐precipitated and digested with trypsin for 4 hr at 42°C in 50 mmol/L ammonium bicarbonate and 50% methanol. The resulting digests were dried *in vacuo* and peptides were resuspended in 0.1% trifluoroacetic acid (TFA) prior to MS analysis. LC/MS analyses were performed using an UltiMate 3000 RSLCnano HPLC system (Thermo Fisher Scientific, Dreieich, Germany) coupled online to a Velos orbitrap Elite instrument (Thermo Fisher Scientific) as described (Hünten et al., 2015) with minor changes for the identification of phosphopeptides. The instrument was operated in the data‐dependent mode using a TOP10 method for the isolation of multiple charged precursor ions. Fragmentation of (phospho)peptides was performed in the linear ion trap using multi‐stage activation with neutral loss masses of 32.7, 49, and 98 Da. An automatic gain control of AGC of 1 × 10^4^ ions and a maximum fill time of 100 ms was used. The dynamic exclusion time window for previously selected precursor ions was 30 s.

For data analysis, MaxQuant (version 1.5.2.8, Cox & Mann, 2008) was used as described (Cristodero et al., 2013) with minor modifications. Briefly, peak lists were searched against a modified UniProt *E. coli* database including the sequences for ArnB, ArnC, and ArnD from *Sulfolobolus acidocaldarius*. Oxidation of methionine, acetylation of protein N‐termini, and phosphorylation of serine, threonine, and tyrosine residues were set as variable modifications. The maximum number of missed cleavages was set to four and the maximum molecular mass to 6000 Da. For relative quantification of (phospho)peptides, intensities of phosphorylated and nonphosphorylated peptides, MS1 intensities were calculated using the Skyline software (version 3.5) (Schilling et al., 2012).

### 2.8 Isolation of total RNA and quantitative reverse transcriptase PCR (qRT‐PCR)

To quantify the mRNA levels of the different genes, quantitative reverse transcriptase PCR was performed**.** 10 ml of a culture was harvested by centrifugation at 3,900*g* for 10 min at 4°C. After removal of the supernatant, the pellet was frozen in liquid nitrogen. Total RNA was isolated using a TRIzol reagent‐based method according to Hottes et al. (2004). Reverse transcription was performed as described by Lassak et al. (2012). The SYBR green Kit for detection (2× qPCRBIO SyGreen Mix Lo‐ROX) was used with the Rotor‐Gene Q Real‐time PCR cycler (Quiagen) according to manufacturer's protocols. Ct values of the analyzed samples were normalized to the housekeeping gene *saci0574* (*secY*). At least three biological and two technical replicates were analyzed.

### 2.9 Isolation of cytosolic and membrane fractions

After determination of the OD_600_, 20 ml of culture was collected and centrifuged at 4,100*g*. For the detection of ArnC and ArnD in the Western blot, cells were collected and the pellet was resuspended in 100 mmol/L KCl, 50 mmol/L HEPES pH 8.0. Subsequently, cells were disrupted by sonification (Bandelin Sonoplus) for 10 min with an intensity of 40%, and a duty cycle of 30 s on and 15 s off and cell debris was removed by low spin centrifugation. The supernatant was used for high spin centrifugation to separate cytoplasm and membranes. Hence, 500 μl low spin supernatant was centrifuged in a TLA‐110 rotor in a Beckman Optima MAX_XP ultracentrifuge at 4°C and 88,000*g* for 30 min. The supernatant (cytoplasm) was collected and directly mixed with 5 x protein loading dye. The pellet (membrane fractions) was resuspended in 150 μl buffer using a homogenizer (Sartorius) and protein‐loading dye was added. The cytoplasm and membranes were used for the detection of ArnC‐HA and ArnD‐HA.

### 2.10 Western blot analysis

For detection of ArnC and ArnD, the cytosolic and membrane fractions were used. For detection of FlaB and FlaX, ArnA and ArnB, samples were taken, centrifuged at 4,100*g*, and resuspended in 100 mmol/L KCl, 50 mmol/L HEPES pH 8.0 to an OD_600_ of 3. Samples were heated to 100°C for 5 min and separated by SDS‐PAGE using 11% gels. Subsequently, proteins were transferred onto PVDF membrane (Roche) applying a semidry method. Equal loading of gels was confirm by staining with InstantBlue (Expedeon). For the detection of FlaB and FlaX (Lassak et al., 2012), ArnA, ArnB (Reimann et al., 2012) primary polyclonal antibodies were used in a dilution of 1:400, 1:800, 1:5,000, and 1:5,000, respectively. Primary antibodies directed against the HA‐tag were obtained from Sigma and used 1:10,000 diluted. As a secondary antibody 1:10,000 diluted goat‐anti‐rabbit immunoglobulin G coupled to HRP (Invitrogen) was applied to the membranes. Chemiluminescent signals were detected with an ECL chemocam imager (Intas) using WesternSure ECL Substrate (LI‐COR). Quantification of Western blots was performed using Image J (Schneider, Rasband, & Eliceiri, 2012).

## 3 Results

### 3.1 Protein kinases are involved in the regulation of motility

**Figure 2:**
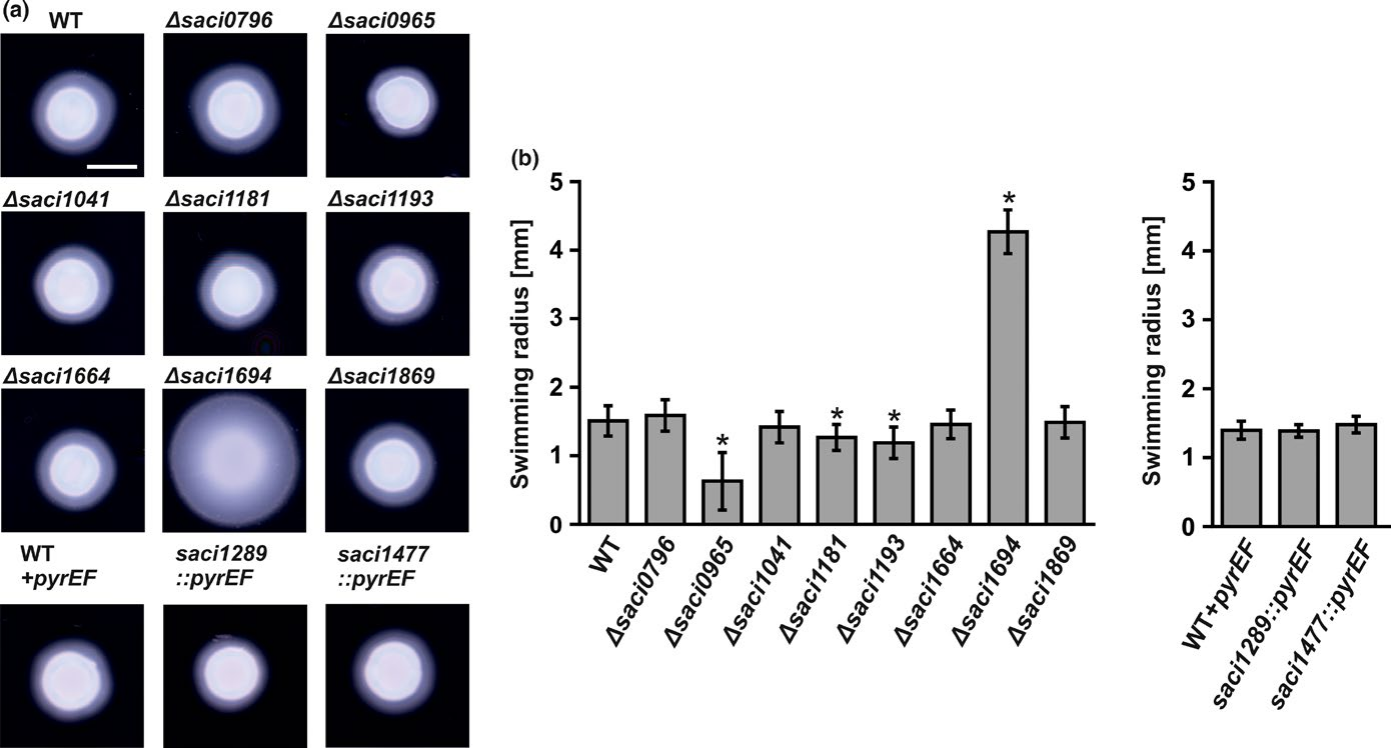
Effect of *S. acidocaldarius* kinase deletion strains on motility. Motility assays were performed on semisolid plates. (a) Motility plates containing the *pyrEF* deletion strain MW001 (WT) or *pyrEF* insertion strain (WT + *pyrEF*) and the kinases deletion strains constructed in the respective background. (b) Average swimming radius calculated from three biological replicates containing each at least six technical replicates. Scale bar 5 mm. Statistical significance for (b) was compared to the WT and WT + *pyrEF* using a Student's t‐test and is indicated by asterisks (*p* ≤ 0.05)

The observation that the deletion of the phosphatase PP2A results in strongly increased motility during starvation and that several of the components of the archaellum regulatory network in *S. acidocaldarius* can be phosphorylated (Reimann et al., 2013), indicated a role for phosphorylation in the regulation of motility. Therefore, we set out to determine which protein kinases might play a role in motility in *S. acidocaldarius*. Based on homology searches, 11 genes encoding putative protein kinases were identified in the genome of *S. acidocaldarius*. Table 1 gives an overview of the predicted kinases of *S. acidocaldarius* with respect to their predicted domains, localization, and the type of kinase. We set‐out to generate deletion mutants of all these protein kinases. Deletion mutants were obtained for all protein kinases except *saci_0850, saci_1289, and saci_1477*. In further attempts, marker (*pyrEF)* insertion mutants could be created for *saci_1289* and *saci_1477*. Neither markerless deletion nor marker insertion mutants could be obtained for *saci_0850*. Saci_0850 is a homolog of the *Saccharomyces cerevisiae* Bud32 kinase, deletion of which resulted in a severe growth retardation (Briza et al., 2002). The obtained mutants were tested for their growth kinetics and in motility assays. For the markerless deletion mutants, the MW001 (WT) strain was used as the control, whereas for the insertion mutants, the MW268 (WT + *pyrEF*) strain which expresses *S. solfataricus pyrEF* was used. Growth assays showed that the mutant strains grew similar to their respective parental strains (Fig. S1). The motility assays were performed by plating cells on plates which contain a reduced amount of gelrite. Motility is only observed on plates with reduced amounts of nutrients, where the expression of the archaellum is induced (Lassak et al., 2012). The deletion of *saci_0965*,* saci_1181,* and *saci_1193* resulted in a significantly reduced motility (Figure 2a and b), whereas the deletion of *saci_1694* resulted in hypermotile cells. In the other deletion strains, no effects on motility could be detected.

**Table 1:** Predicted protein kinases of *S. acidocaldarius*

Protein	Transmembrane domains	Conserved domains	Additional information
Saci0796	No	Rio 2 catalytic domain	Atypical PK, predicted RIO 2 kinase
Saci0850	No	PK domain, Rio 1 domain	Atypical PK, Bud32 homolog (RIO‐type kinase), subunit of KEOPS complex
Saci0965	No	RIO catalytic domain	Atypical PK, predicted RIO 1 kinase
Saci1041	Yes, 5 (N‐terminal)	PK domain	Typical PK
Saci1181	Yes, 2 (N‐terminal)	PK domain	Typical PK, predicted Ser/Thr‐specific
Saci1193	No	PK domain, TPR domain	Typical PK, Ser/Thr‐specific
Saci1289	No	PK domain	Typical PK
Saci1477	No	PK domain	‐
Saci1664	Yes, 1 (C‐terminal)	Rio 1 catalytic domain, ABC1 domain	Atypical PK, predicted ABC1 family kinase
Saci1696	No	PK domain	Typical PK, Ser/Thr‐specific
Saci1869	Yes, 5 (N‐terminal)	PK domain, coiled coil domain	Typical PK

Domain structure and additional information was obtained from SMART, uniprot, and the conserved domain database on NCBI (Marchler‐Bauer et al., 2015; Schultz, Milpetz, Bork, & Ponting, 1998; UniProt Consortium, 2015).

To identify possible targets of the protein kinases involved in the regulation of the archaellum, we set‐out to determine whether the presence of orthologs of these kinases in different genomes was connected to the occurrence of the archaellum. Since regulatory pathways might differ between crenarchaea and euryarchaea, the analysis focused on the crenarchaea. First, the presence of the operon encoding the archaellar proteins, and the presence of currently identified proteins involved in regulation of the archaellum, (ArnA, ArnB, ArnR, ArnR1, and AbfR1) was determined in the 26 species (Fig. S2). The archaellum operon was identified in 11 of the 26 genomes. None of the currently identified regulators of the archaellum operons, was identified in all genomes that contained the archaellum operon. Analysis of the regions flanking the archaellum operons did not identify regulatory elements conserved in all organisms containing the archaellum operon, suggesting that the other archaellum operons are either not regulated or regulated in a different manner. All studied genomes of Sulfolobales containing the archaellum operon also encoded one ortholog of the positive regulators ArnR/ArnR1. Only in *S. acidocaldarius*, highly homologous ArnR and ArnR1 are both present suggesting that they derived from a duplication event (Lassak et al., 2013). No orthologs of ArnR/ArnR1 could be identified in genomes that did not contain an archaeallum operon, suggesting that ArnR/ArnR1 is specifically involved in regulation of the archaellum (Lassak et al., 2013). Orthologs of the repressors ArnA and ArnB, and of AbfR1 were identified in all analyzed Sulfolobales genomes. Thus also in the genomes of *Acidianus hospitalis* W1 and *Metallosphaera cuprina*, that did not contain an archaellum operon, suggesting that, next to their role in the regulation of the archaellum in *S. acidocaldarius*, these proteins also fulfill additional roles in nonarchaellated creanarchaea.

Analysis of the occurrence of orthologs of the four protein kinases for which deletion had an effect on motility, showed that orthologs of *saci_0965* were identified in all included crenarchaea, whereas orthologs of *saci_1193* were restricted to the Sulfolobales, orthologs of *saci_1181* were restricted to Sulfolobus strains, and no orthologs of s*aci_1694* were identified outside *S. acidocaldarius*. The occurrence of none of these kinases could be directly linked to the presence of the archaellum operon, but orthologs of *saci_1193* could be identified in all Sulfolobales that contained the repressors ArnA and ArnB. We have previously shown that Saci_1193 phosphorylates ArnA and ArnB, whereas Saci_1694, phosphorylates ArnB, but not ArnA (Reimann et al., 2012). ArnA and ArnB play an important role in the regulation of the expression of components of the archaellum (Reimann et al., 2012), suggesting that Saci_1193 and Saci_1694 might also be involved in this process. Orthologs of Saci_0965 were found in all studied crenarchaea. Saci_0965, which belongs to the atypical PKs is the Rio 1 kinase homolog of *S. acidocaldarius*. The Rio 1 kinases are involved in ribosome biogenesis in eukaryotes and the deletion of Rio2 in *S. cerevisieae* is not viable (Laronde‐LeBlanc & Wlodawer, 2005a; LaRonde‐LeBlanc & Wlodawer, 2005b). Interestingly, we were able to obtain a viable deletion mutant. However, since this gene is also found in all crenarchaeal chromosomes that do not contain the archaellum operon, a more general role is expected for this protein kinase, and we did not focus on it in this study. We therefore, decided to focus our study on the role of Saci_1193 and Saci_1694 in motility, and propose to name them ArnC and ArnD, respectively. The characterization of Saci_1181 is described elsewhere (Haurat et al., unpublished).

### 3.2 ArnC and ArnD function in a complex concentration‐dependent manner

**Figure 3:**
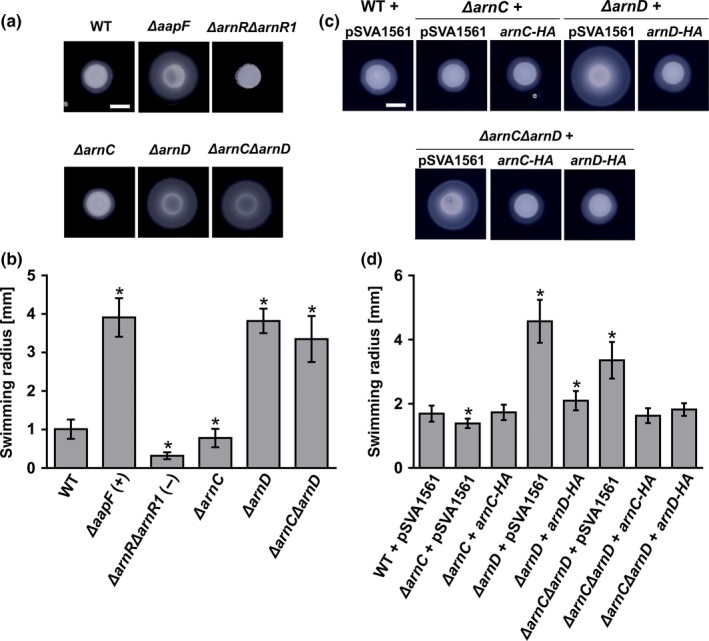
*arnC* and *arnD* deletion mutants are affected in motility. Motility assay were performed on semisolid plates. (a) Motility plates containing the *pyrEF* deletion strain MW001 (WT) and MW001 derivatives containing *ΔaapF*,* ΔarnRΔarnR1*,* ΔarnC, ΔarnD, and ΔarnCΔarnD*. (b) Average swimming radius calculated from three biological replicates containing each at least six technical replicates. (c) Motility assay of the strains shown in (a). carrying either pSVA1561 (*S. acidocaldarius* expression vector) or pSVA1561 containing *arnC‐HA* or *arnD‐HA* under the control of their native promoters. HA indicates a C‐terminal HA‐tag. (d) Average swimming radius calculated from three biological replicates containing each at least six technical replicates. Scale bar; 5 mm. Statistical significance for (b) and (d) was compared to the WT using a Student's t‐test and is indicated by asterisks (*p* ≤ 0.05)

The deletion of *arnC* and *arnD* results in opposite motility phenotypes. The WT and the *ΔarnC* and the *ΔarnD* mutants were studied by electron microscopy (EM), and all three strain assembled archaella (See Fig. S4). We did not observe a difference in the number of archaella per cell between the WT and the *ΔarnC* and the *ΔarnD* mutants (~1–2 archaella per cell). The number of cells on which archaella were observed after imaging seemed to be lower in the *ΔarnC* mutant, whereas the *ΔarnD* mutant showed a higher number of cells which contained an archaellum. In order to understand how the function of the *arnC* and *arnD* genes intersect, a double deletion mutant was created, and motility assays were performed (Figure 3a and b). A hypermotile (Δ*aapF*) and a nonmotile strain (Δ*arnR*/Δ*arnR1*) were included (Henche et al., 2012; Lassak et al., 2013). The motility radius of the *ΔarnC* strain was again ~ 30% smaller than the radius observed for the wild‐type cells, and deletion of *arnD* resulted in hypermotile cells. The double mutant also showed a hypermotile phenotype, and was only slightly less motile than the Δ*arnD* strain, demonstrating that the *arnC*/*arnD* double mutant mostly phenocopies the *arnD* deletion mutant. Furthermore, to exclude any polar effects of the created single or double mutants, plasmids were created that contained *arnC* and *arnD* and their respective upstream promoter regions. The plasmid pSVA1561 expressing the β‐galactosidase gene, *lacS* from *S. solfataricus* was used as a backbone for construction of the kinase expression plasmids. Since no antibodies against ArnC and ArnD are available, sequences encoding C‐terminal HA‐tags were included in these vectors to enable us to detect the proteins. Strains carrying pSVA1561 showed a similar trend in motility as observed before for the strains without the plasmid (Figure 3c and d). The *ΔarnC* carrying pSVA1561 was about 20% less motile than the WT strain carrying pSVA1561, and the *ΔarnD* strain carrying pSVA1561 was hypermotile. When introducing the plasmids carrying the respective kinases, ArnC‐HA expression complemented the motility of the *ΔarnC* mutant to WT level. Expression of ArnD‐HA resulted in a significantly decrease in the hypermotility phenotype, and almost completely restored the WT phenotype, demonstrating that the observed phenotypes were not caused by any polar effects. Analysis of the *arnC*/*arnD* double deletion strain in the presence of the pSVA1561 plasmid showed that the strain was again hypermotile, but remarkably was less motile than the *arnD* single mutant (Figure 3c and d). The difference between the motility of the *arnC*/*arnD* double deletion strain and the *arnD* single mutant in the presence (Figure 3a and b) and absence (Figure 3c and d) of the pSVA1561 plasmid might be caused by the increased growth rate of the strain in the presence of *pyrEF* containing plasmids and the higher copy number of the kinase encoding gene (Berkner, Wlodkowski, & Lipps, 2010). The observation that under these conditions, the *arnC*/*arnD* double deletion strain is less mobile than the *arnD* single mutant shows that *arnC and arnD* fulfill complementary functions. Interestingly, the phenotype of the double deletion was fully complemented by the expression of either ArnC‐HA or ArnD‐HA. Thus although the deletion mutants of the *arnC* and *arnD* show opposite motility phenotypes, (over)‐expression of either protein can complement the motility phenotype of the *arnC*/*arnD* double mutant, suggestion a complex regulatory mechanism that depends on the exact concentrations of the two kinases.

#### 3.2.1 ArnC and ArnD are Ser/Thr kinases that phosphorylate ArnB on its C‐terminus

**Figure 4:**
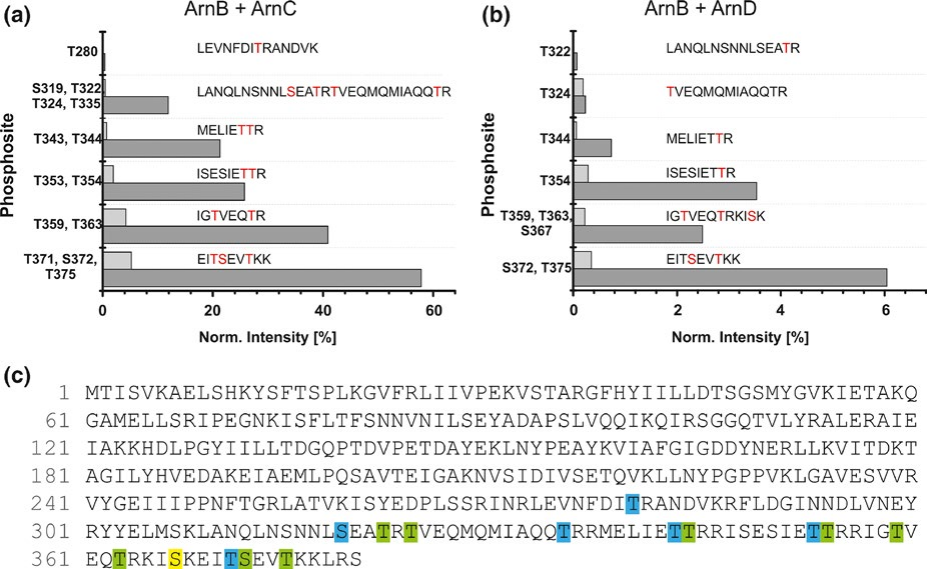
ArnB is phosphorylated by ArnC and ArnD on serine and threonine residues. (a) 1 μg of ArnB was incubated with 20 ng (light gray bars) or 2 ng (dark gray bars) ArnC or (b) ArnD, and phosphorylated peptides were determined by mass spectrometry. Normalized intensities of different phosphopeptides are depicted. The average of three replicates is depicted (c) Sequence of ArnB annotated with phosphorylation sites detected with ≥75 % probability. Residues detected after phosphorylation with ArnC only (blue), ArnD only (yellow) and the ones that were detected in both assays (green) are highlighted. The annotated MSMS spectra and tables used for the calculation of the phosphorylation intensity are available as Supporting file 1 (pdf: Annotated MSMS spectra of phosphorylated ArnB peptides) and Supporting file 2 (excel file: Quantitative MS data from in vitro kinases assays)

ArnC and ArnD belong to the family of ePKs, which generally phosphorylate serine, threonine and/or tyrosine residues. ArnD consists of only the conserved kinase domain, but the kinase domain of ArnC is preceded by two tetratricopeptide repeat motifs (TPR) ((Fig. S2 A) (Lamb, Tugendreich, & Hieter, 1995). TPR motifs are normally involved in facilitating protein‐protein interactions. Sequence alignments and modeling showed that ArnC and ArnD contains the 12 typical subdomains found in ePKs (Fig. S2 B and S2 C). Previously, it was demonstrated that incubation of stoichiometric amounts of ArnC or ArnD with ArnB resulted in phosphorylation of ArnB (Reimann et al., 2012). In order to determine the specificity of ArnC and ArnD, *in vitro* phosphorylation assays were performed with ArnB at lower ArnC and ArnD concentrations. 1 μg of ArnB was incubated in the presence of Mn^2+^ and ATP with 20 ng (50‐times less) and 2 ng (500‐times less) ArnC or ArnD in a buffer that was optimized for the activity of the respective kinase. After the incubation and treatment with trypsin, the presence of phosphorylated peptides was determined by mass spectrometry (Figure 4). Analysis of the most abundant phosphopeptides demonstrated that both ArnC and ArnD phosphorylate ArnB specifically at serine and threonine residues in its C‐terminus, and no phosphorylation of tyrosine residues was observed (Figure 4c). Phosphorylation was observed even at low amounts of the kinase compared to the ArnB substrate, demonstrating that the phosphorylation is specific. Approximately 10‐fold less phosphorylation of ArnB was observed for ArnD compared to ArnC. Identification of the sites targeted by ArnC and ArnD showed that the phosphorylation patterns strongly overlapped. In a study where phosphorylated peptides were identified in cell extracts from an exponentially growing *S. acidodaldarius* strain, four phosphorylated residues (T280, T343, T344, and T353) were detected in ArnB (Reimann et al., 2013). Phosphorylation of T344 was found after incubation with either kinase, and phosphorylation of T280, T343, and T353, was identified after incubation with ArnC (Figure 4c). Thus all residues that were found to be phosphorylated in the *in vitro* study were also found in our *in vitro* assay. Thus ArnC and ArnD phosphorylate ArnB even at low concentrations specifically at serine and threonine residues at the C‐terminus. In summary, these data suggest that ArnC and ArnD function as Ser/Thr‐specific kinases that phosphorylate ArnB at various residues *in vitro*. Hence, both kinases might influence expression of other components of the archaellum regulatory network, too.

#### 3.2.2 *arnD*, but especially *arnC* is upregulated during starvation

**Figure 5:**
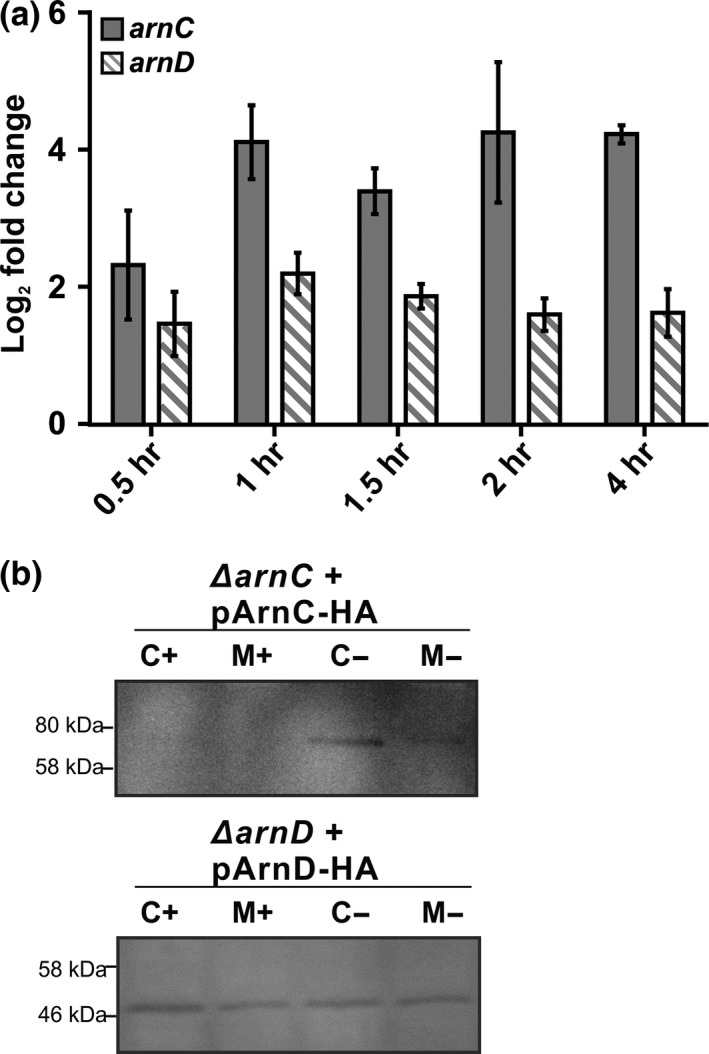
Analysis of the expression of *arnC* and *arnD* during growth on rich and starvation medium. **(**a) Samples of *S. acidocaldarius* were grown in rich medium, centrifuged and then transferred to either rich or starvation conditions and mRNA levels of *arnC* and *arnD* and were determined by qRT‐PCR at different time‐points. The increase in expression in the starvation medium compared to the rich medium is shown. Bars show the mean value of three independent biological replicates with each four technical replicates. (b) Western blot of the levels of ArnC‐HA and ArnD‐HA in the cytoplasm (C) and the membrane (M) after 4 hr of growth under rich (+) and starvation (‐) conditions. The membranes were concentrated 3.3‐fold compared to the cytoplasmic fraction. Proteins were detected using an antibody directed against the HA‐tag. The figure shows a representative blot of three independent replicates

Since deletion of *arnC,* but especially *arnD* affected the motility of *S. acidocaldarius*, we set out to determine if *arnC* and *arnD* are regulated during starvation. To this end, a *S. acidodaldarius* culture was grown in rich medium, centrifuged and resuspended in basal Brock medium, either with (rich conditions) or without 0.1% NZamine and 0.2% dextrine (starvation conditions) and samples were taken over a 4 hr period. Two‐step quantitative reverse transcription PCR (qRT‐PCR) was performed to determine the change in mRNA levels of *arnC* and *arnD* (Figure 5a). The relative gene expression of *arnC* was about 16‐fold upregulated under starvation conditions as compared to nutrient‐rich conditions, whereas *arnD* was only two to fourfold upregulated. To test whether these trends are reflected in the amounts of protein as well, the levels of ArnC and ArnD in the cytoplasm were determined 4 hr after either growth under rich or starvation conditions (Figure 5b). Since no antibodies against ArnC and ArnD are present, the ArnC‐HA and ArnD‐HA levels were determined in the *ΔarnC* and *ΔarnD* strains expressing plasmid‐encoded *arnC‐HA* and *arnD‐HA* from their own promotor. In good agreement with strong upregulation of *arnC*, and the much lower upregulation of *arnD*, ArnC‐HA levels increased under starvation conditions to a detectable level, whereas ArnD could be detected at similar levels in rich conditions as under starvation conditions. Since many components of the archaellum and its regulatory network are membrane bound, the levels of ArnC‐HA and ArnD‐HA in membranes were also determined (Figure 5b). No ArnC‐HA could be detected in the membrane fraction, but approximately 25% of the ArnD‐HA was associated with the membrane, suggesting a possible interaction of ArnD with a membrane‐bound component. Similar to what was observed for the cytosolic ArnD, no change in the membrane‐bound levels of ArnD was observed between the rich and the starvation conditions. Thus, the expression and protein levels of ArnC are increased during starvation, whereas ArnD seem be present both during rich and starvation conditions.

#### 3.2.3 FlaB levels are increased in the Δ*arnD* strain during growth in rich medium

**Figure 6:**
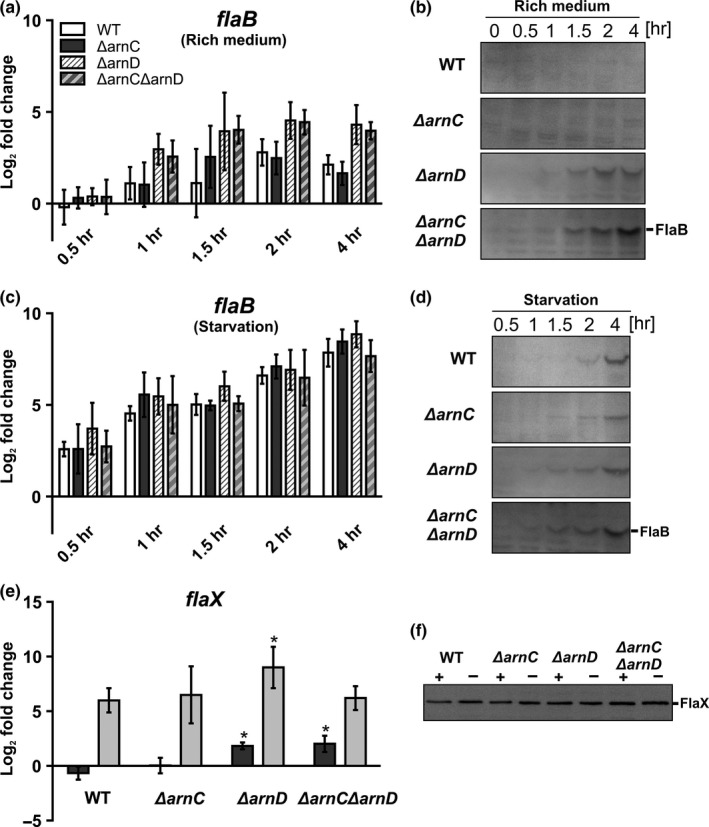
Expression and protein levels of FlaB and FlaX in rich and starvation medium. *S. acidodaldarius* was grown to an OD_600_ of 0.4 in rich medium and transferred to either rich or starvation medium and samples for total RNA extraction and Western blot were collected. (a) Relative gene expression levels of *flaB* after 0.5 hr, 1 hr, 1.5 hr, 2 hr and 4 hr growth in rich medium compared to *t* = 0 of the WT (white bars) and the *ΔarnC* (gray bars), *ΔarnD* (light gray, dashed bars), and *ΔarnCΔarnD* (dark gray, dashed bars) strains. (b) Western blots of FlaB in whole cells grown in rich medium. Proteins were detected using an antibody directed against FlaB. A representative blot of at least three independent replicates is shown. (c) Relative gene expression levels of *flaB* after 0.5 hr, 1 hr, 1.5 hr, 2 hr and 4 hr growth in starvation medium compared to *t* = 0 of the WT (white bars) and the *ΔarnC* (gray bars), *ΔarnD* (light gray, dashed bars), and *ΔarnCΔarnD* (dark gray, dashed bars) strains. (d) Western blots of FlaB in whole cells grown in starvation medium. Proteins were detected using an antibody directed against FlaB. A representative blot of at least three independent replicates is shown. (e) Relative gene expression levels of *flaX* after 4 hr of growth in rich medium (dark gray bars) and starvation medium (light gray bars) compared to *t* = 0 of the WT and the *ΔarnC*,* ΔarnD,* and *ΔarnCΔarnD* strains. (f). Western blots of FlaX in whole cells grown in starvation medium and rich medium for 4 hr. Proteins were detected using an antibody directed against FlaX. A representative blot of at least three independent replicates is shown. Bars in (a), (c), and (e) represent the mean values of three independent biological replicates with each four technical replicates. Values that are significantly different compared to the WT (Student's t‐test, *p* ≤ 0.05) are indicated by asterisks

To understand how ArnC and ArnD influence motility, we analyzed the expression and protein levels of *flaB* in the *ΔarnC*,* ΔarnD,* and *ΔarnCΔarnD* strains during growth on rich or under starvation conditions. To this end, the wild type and kinase deletion mutants were grown in rich medium and after reaching an optical density of 0.4, the cultures were split in two and pelleted (*t* = 0). Subsequently, the cells were resuspended in either rich or starvation medium and grown for another 4 hr. Samples were taken at different times. From these cells, the amounts of *flaB* mRNA relative to their amounts at *t* = 0 were determined via qRT‐PCR (Figure 6a and c). Furthermore, the protein levels of FlaB were determined via Western blotting (Figure 6b and d). In the WT strain, the mRNA levels of *flaB* increased fourfold after growth on rich medium for 4 hr, but the FlaB protein could not be detected under these conditions. As described previously, after 4 hr of growth on starvation medium, *flaB* levels in the WT strain were strongly increased (~ 240‐fold) and the FlaB protein was easily detected (Lassak et al., 2012). Furthermore, archaella were observed in the wild‐type *S. acidocaldarius* (Fig. S4).

Contrary to what we expected, no strong changes were observed in the *flaB* mRNA levels between the WT and the *ΔarnC*,* ΔarnD,* or *ΔarnCΔarnD* strains after growth on starvation medium. FlaB could be detected slightly earlier in the *ΔarnD* or *ΔarnCΔarnD* strains. Remarkably, a much larger difference was observed between the WT and *ΔarnC*, and the *ΔarnD* and *ΔarnCΔarnD* strains during growth on rich medium. After 1 hr of growth of the *ΔarnD* and *ΔarnCΔarnD* on rich medium expression of *flaB* in the *ΔarnD* and *ΔarnCΔarnD* strains was increased, and FlaB was also detected. Therefore, the strong effect of *arnD* deletion observed on motility plates is most likely not only caused by an effect on the expression levels of FlaB or the archaellum complex.

Since the protein levels of FlaX also depend on the functional expression of other proteins in the archaellar complex like FlaH, FlaX levels can function as an indicator for functional assembly of the archaellum (Chaudhury et al., 2016). The mRNA levels of *flaX* did not change after growth on rich medium, but, after growth on starvation medium, higher amounts of *flaX* mRNA were detected (Figure 6e). However, in repeated experiments, no significant differences could be detected between the FlaX protein levels after either growth on rich or starvation medium (Figure 6f). Indeed this confirms that FlaB is under control of a promotor that is induced under starvation condition, whereas *flaX‐flaJ* are under control of a constitutive promotor. The increase in *flaX* mRNA levels might be caused by read‐through from the promotor before *flaB*, but increase in this mRNA does not result in an increase the FlaX protein levels (Lassak et al., 2012).

This suggests a possible modulation of the activity of the archaellum via ArnD. However, after growth on rich medium, both the mRNA and protein levels of FlaB were significantly increased in the *ΔarnD* strain. Remarkably, even higher levels of FlaB were found in the *ΔarnCΔarnD* strains. Thus only after growth of the *ΔarnD* and *ΔarnCΔarnD* strains under rich conditions, differences in the FlaB protein levels could be detected.

#### 3.2.4 Expression of regulators of the archaellumand genes of the archaeal adhesive pilus are not changed in the Δ*arnC*, Δ*arnD* or Δ*arnC*Δ*arnD* strains

**Figure 7:**
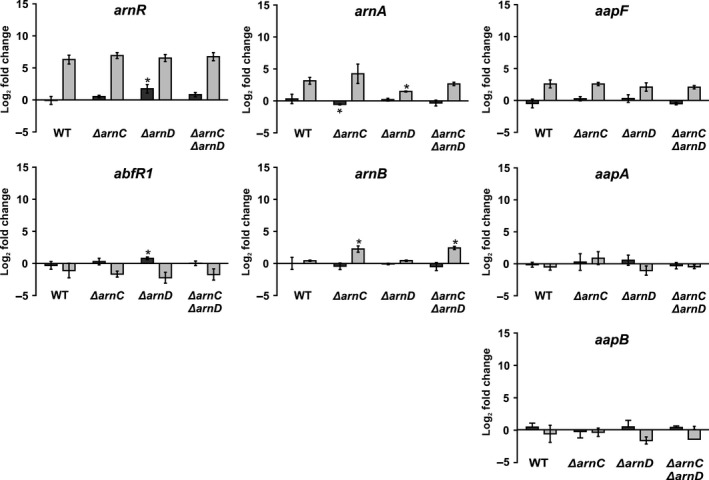
Expression of archaellum regulators and adhesive pilus genes in the WT and *ΔarnC*,* ΔarnD,* and *ΔarnCΔarnD* strains. Total RNA was extracted from cells that were grown to an OD_600_ of 0.4 and subsequently shifted to either rich or starvation medium. Relative gene expression levels of *arnR*,* abfR1*,* arnA*,* arnB*,* aapA,* and *aapF* in rich (dark gray bars) and starvation medium (light gray bars) compared to *t* = 0 in each strain. Bars represent mean values of three independent biological replicates with each four technical replicates. Values that are significantly different compared to the WT (Student's t‐test, *p* ≤ 0.05) are indicated by asterisks

Since, in the *ΔarnD* or *ΔarnCΔarnD* strains, we observed upregulation of FlaB after growth on rich medium, and a hypermotile phenotype under starvation conditions, we set out to see if any of the other known regulator were regulated differently in the *ΔarnC*,* ΔarnD* or *ΔarnCΔarnD* strains. Therefore, the mRNA levels of *arnA*,* arnB*,* arnR,* and *abfR1* were determined (Figure 7a–d). Small changes were observed under several conditions (e.g., *arnR* levels are increased in the *ΔarnD* strain in rich medium, and *arnB* levels are increased in the *ΔarnC* and *ΔarnCΔarnD* mutants in starvation medium). However, the changes in the expression levels of these regulators were relatively small compared to the differences observed between growth on rich and on starvation medium, and for example, increase in *arnR* levels observed in the *ΔarnD* strain in rich medium, are not observed for the *ΔarnCΔarnD* strains, where we also find upregulation of *flaB*. Therefore, we were unable to link the small changes in the expression levels of the regulators to the observed hypermotility phenotype in starvation medium, or to the increased expression levels of *flaB* on rich medium.

Expression and function of the archaellum and the archaeal adhesive pilus (aap) are strongly linked. Interestingly, overexpression of ArnA results in a strong increase in aap pili on the surface of *S. acidocaldarius*. Furthermore, the deletion of *aapF* leads to upregulation of *flaB*, and a hypermotility phenotype (Henche et al., 2012; Reimann et al., 2012). Additionally, electron microscopy pictures suggest that the arnC deletion mutant assembled more aap pili than the wild type and the *ΔarnD* cells seem to have less aap pili than both wild type and *ΔarnC* cells (Fig. S4). Therefore, we also tested the changes in the expression of *aapF (membrane protein)* and *aapA* as well as *aapB* (pilins) in wild type and *ΔarnC*,* ΔarnD,* or *ΔarnCΔarnD* strains after growth in rich and starvation medium (Figure 7e and f). However, no significant differences were observed when the deletion mutants were compared to the WT suggesting that a connection between the aap pilus and motility is not regulated on a transcriptional level but rather hints to a posttranslational mechanism.

## 4 Discusssion

When nutrients become limiting, *S. acidocaldarius* can use the archaellum, to move to more favorable surroundings. The expression and activity of the archaellum in *S. acidocaldarius* is strictly controlled by a sophisticated regulatory network. Since the deletion of the phosphatase PP2A results in strongly increased motility during starvation, and several of the components of the archaellum regulatory network in *S. acidocaldarius* can be phosphorylated, an important role for phosphorylation in the regulation of motility was proposed (Lassak et al., 2013; Reimann et al., 2012). Surprisingly little is known about phosphorylation in archaea even though most of the sequenced genomes encode typical and atypical ePKs as well as protein phosphatases (Esser & Siebers, 2013; Esser et al., 2011). The biochemical properties of ePKs of *S. solfataricus*,* S. tokodaii,* and *S. acidocaldarius* were characterized with respect to their kinase activities (Haile & Kennelly, 2011; Lower & Kennelly, 2002, 2003; Lower et al., 2000, 2004; Ray et al., 2015), but still little is known about which proteins are the natural targets of the kinase and how these proteins function *in vivo*. Here, we set out to identify possible protein kinases that play a role in the regulation of motility. Up to now no two‐component systems have been identified in crenarchaea (Ashby, 2006), but 11 putative Hanks‐type or eukaryotic protein kinases (ePKs) were identified in *S. acidocaldarius*. The availability of methods to generate *pyrEF* insertion and markerless deletion mutants enabled us to create 10 deletion mutants of protein kinases (Orell et al., 2013; Wagner et al., 2012). Only the deletion mutant of *saci_0850*, a homolog of the Bud32 subunit of the *S. saccharomyces* KEOPS complex involved in tRNA modifications was not obtained. Analysis of the motility of the ten protein kinase deletion strains revealed that the deletion of *saci_0965*,* saci_1181*, and *arnC* resulted in reduced motility, whereas the deletion of *arnD* resulted in hypermotility. This further confirms that phosphorylation plays an important role in the regulation of motility in *S. acidocaldarius, and* that four kinases might play important roles in this regulation.

The kinase Saci_0965 belongs to the so called RIO kinases, a group of ubiquitous atypical kinases comprised of four subfamilies, Rio 1, Rio 2, Rio 3, and Rio B (Laronde‐LeBlanc & Wlodawer, 2005a; LaRonde‐LeBlanc & Wlodawer, 2005b). In eukaryotes, like *S. cerevisiae*, these kinases are involved in processes like cell cycle progression and maturation of the small ribosome subunit, which are crucial for survival and consequently, RIO kinases are essential in eukaryotes (Laronde, 2014; Laronde‐LeBlanc & Wlodawer, 2005a; LaRonde‐LeBlanc & Wlodawer, 2005b). Interestingly, we could obtain deletion mutants of both RIO kinases of *S. acidocaldarius*, suggesting that here these genes are not essential for survival but might affect growth nonetheless. This will be subject of further studies. In addition to Saci_0965 also deletion of the membrane‐bound protein kinase Saci_1181 resulted in a reduced motility. Saci_1181 is encoded in relative proximity of the archaellum operon and its characterization is described elsewhere (Haurat et al., unpublished).

Here, the ArnC and ArnD kinases were further characterized. It was previously demonstrated that incubation of stoichiometric amounts of ArnC or ArnD with ArnB resulted in phosphorylation of ArnB (Reimann et al., 2012), and here we demonstrated that ArnC and ArnD specifically phosphorylate serine and threonine residues in the C‐terminus of the repressor ArnB. Meanwhile, several of the residues phosphorylated by ArnC and ArnD were also identified in a study of the phosphoproteome of *S. acidocaldarius*, confirming that phosphorylation of these residues also occurs in vivo (Reimann et al., 2013). The observation that phosphorylation by ArnC and ArnD is specific for serine and threonine residues fits with the observation that motility seems to be unaffected by deletion of Saci_PTP, the only phosphatase that dephosphorylates tyrosine residues. Interestingly, some of the identified residues lie within motifs that could be involved in the interaction of ArnB with ArnA, suggesting that phosphorylation might influence the interaction between ArnA and ArnB.

It is, however, remarkable that, despite the mostly overlapping phosphorylation sites of ArnC and ArnD, both deletion mutants showed opposite motility phenotypes. Indeed, in the strains that contain the empty vector (pSVA1561), combination of the *arnC* and *arnD* deletions reduced the hypermotility phenotype of the *arnD* deletion. Interestingly, the hypermotility phenotype of the *arnC/arnD* deletions strain could also be fully complemented by overexpression of *arnC*. Thus, the hypermotility phenotype of the *arnD* deletion strain can be complemented by both deletion and by overexpression of *arnC*. This suggests a complicated regulatory network in which the specific concentrations of ArnC and ArnD seem to play a crucial role.

A comparable increase in motility, as was observed for the *ΔarnD* strain, was previously observed for deletions of the negative regulators ArnA and ArnB as well as of the PP2A phosphatase (Reimann et al., 2012, 2013). In these deletion mutants, a clear increase in the expression levels of FlaB was observed compared to the WT strain. Indeed, in the *ΔarnD* strain, both when grown on rich and on starvation medium, FlaB accumulated earlier than the WT strain. However, after 4 hr starvation, the FlaB expression levels do no longer differ between the *ΔarnD* and the WT strains. In this respect, also no effects were observed on the expression of the repressors *arnA* and *arnB*, the activators *arnR* and *abfR1* or the components of the Aap pilus. Thus the *ΔarnD* hypermotility phenotype observed on plates under starvation conditions, is under conditions where the FlaB levels are most likely no longer significantly different from the expression levels observed for the WT. Thus ArnD might play a role in the initial phase of the expression of *flaB*, but most likely also plays a second currently unidentified role. A similar conclusion might be drawn for ArnC. Although, a significant decrease in motility was observed, no differences in the *flaB* or *flaX* levels were visible between the WT and the *ΔarnC* strain. Thus, ArnC also might have a second currently unidentified role.

Possible processes that might be affected by the ArnC and ArnD kinases are the switch between assembly and rotation and a possible direct regulation of the activity of the archaellum. The protein that forms the inner membrane assembly platform of the archaellum, FlaJ, was phosphorylated in vivo at several sites in exponential growth phase (Reimann et al., 2013). Possibly other cytoplasmic components like, for example, FlaH and FlaX can be phosphorylated causing the switch from rotation to assembly or the activity of the achaellum. To identify the proteins via which ArnC and ArnD execute their function should be aim of future studies.

These studies might also address a possible correlation between ArnD and the Aap pilus. Interestingly, a functional Aap pilus was, similar to ArnD, up to now only identified in *S. acidocaldarius*. This pilus is necessary for attachment of the cells to surfaces and involved in biofilm formation (Henche et al., 2012). The *aap* pilus and the archaellum are thus important for the sessile and motile lifestyles, respectively. Indeed it was shown that the deletion of most of the *aap* pilus genes, especially the ATPase *aapF*, result in hypermotile cells with high *flaB* expression (Henche et al., 2012; Lassak et al., 2012). Moreover; the deletion of the repressor *arnA* resulted in a strong increase in *aap* pili on the surface of the cells (Reimann et al., 2012). Interestingly, our EM images, suggested that more aap pili could be observed on the surface of *ΔarnC* cells than on WT cells, whereas less aap pili were observed on the surface of *ΔarnD* cells (Fig. S4). Since the expression levels of all tested aap pili genes were not significantly changed between WT and kinase deletion mutants (Figure 7), it is possible that the kinases ArnC and ArnD are involved in the regulation of the aap pilus and the archaellum in a complex, possibly posttranslational, yet unknown manner. A connection between pili and motility was also demonstrated in *Haloferax volcannii*. Here it was shown that the deletion of the adhesive pilins (PilA1‐6) caused severe motility defects and the authors suggested that a specific domain of these pilins (H‐domain) is needed for motility regulation in a posttranslational mechanism which is not dependent on the formation of a functional pilus (Esquivel & Pohlschroder, 2014).

Thus, we were able to show that two Ser/Thr‐specific kinases of *S. acidocaldarius* are involved on several levels within the complex regulatory mechanism ultimately leading to a fully functional archaellum and therefore, motility. It will be interesting to find the connection of the kinases to known or yet unidentified components of the archaellum regulatory network, the archaellum itself and maybe even the *aap* pilus of *S. acidocaldarius*.

## Conflict of Interest

None declared.
