# Supplementary material for: Expanding the archaellum regulatory network – the eukaryotic protein kinases ArnC and ArnD influence motility of Sulfolobus acidocaldarius
Source: Microbiologyopen. 2016 Oct 22;6(1):e00414. doi: 10.1002/mbo3.414 (PMC5300886; doi:10.1002/mbo3.414)
Supplement: Supplementary file 1 [file MBO3-6-0-s001.docx]

**Supporting Information to:**

**Expanding the archaellum regulatory network – the eukaryotic protein kinases ArnC and ArnD influence motility of *Sulfolobus acidocaldarius***

Lena Hoffmann, Andreas Schummer, Julia Reimann, M. Florencia Haurat, Amanda J. Wilson, Morgan Beeby, Bettina Warscheid and Sonja-Verena Albers

**
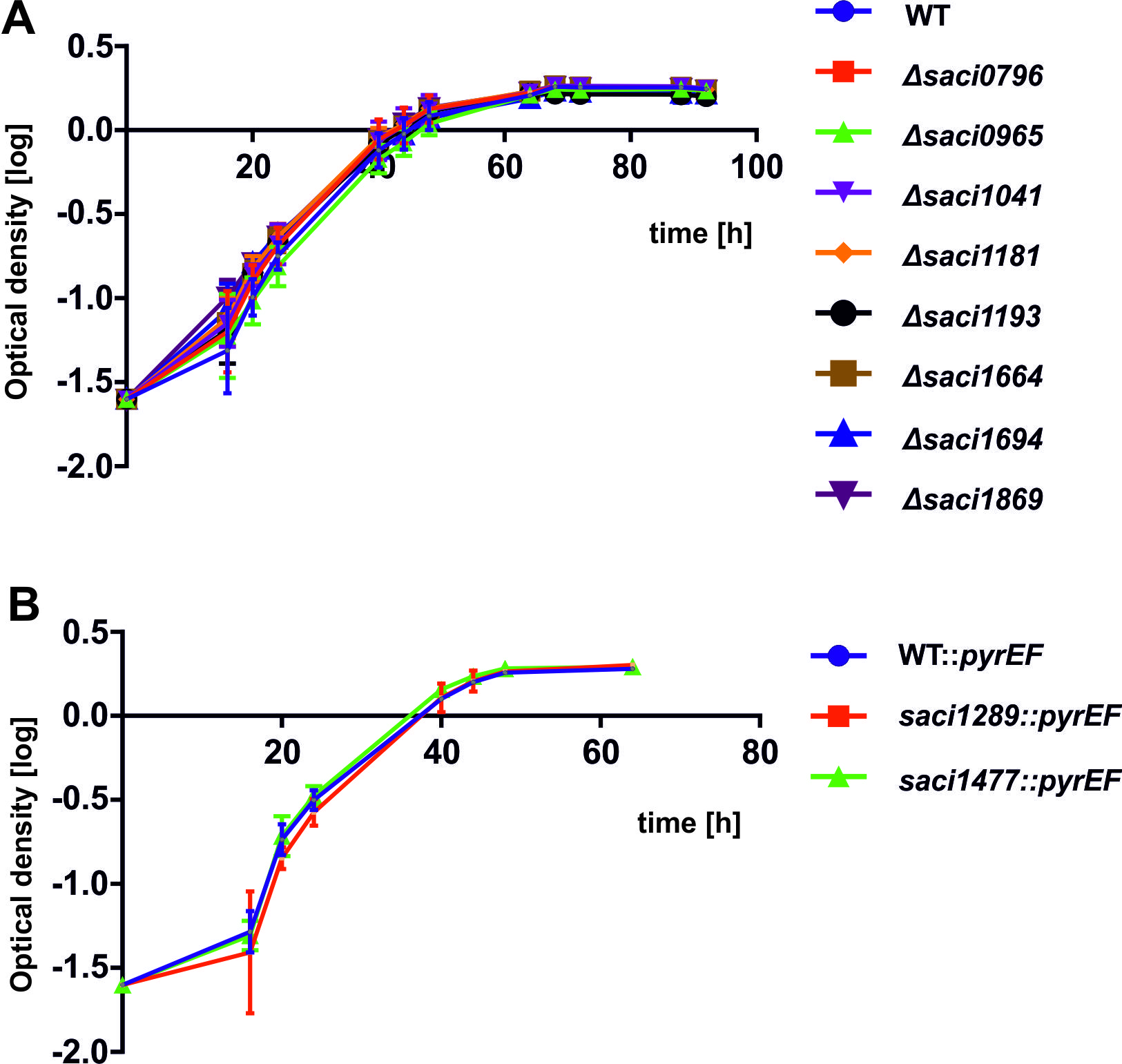
**

**Fig. S1. Growth curves of the kinase mutants on NZ-amine and dextrin.** Optical density at 600 nm of kinase mutants and the respective WT strains were monitored in 5 ml cultures over time until stationary growth phases was reached. For each strain two biological replicates with three technical replicates were measured, converted to log scale and mean values including standard deviations were plotted in graphs for the WT and the clean deletion mutants (A) and *pyrEF* insertion mutants (B).

**
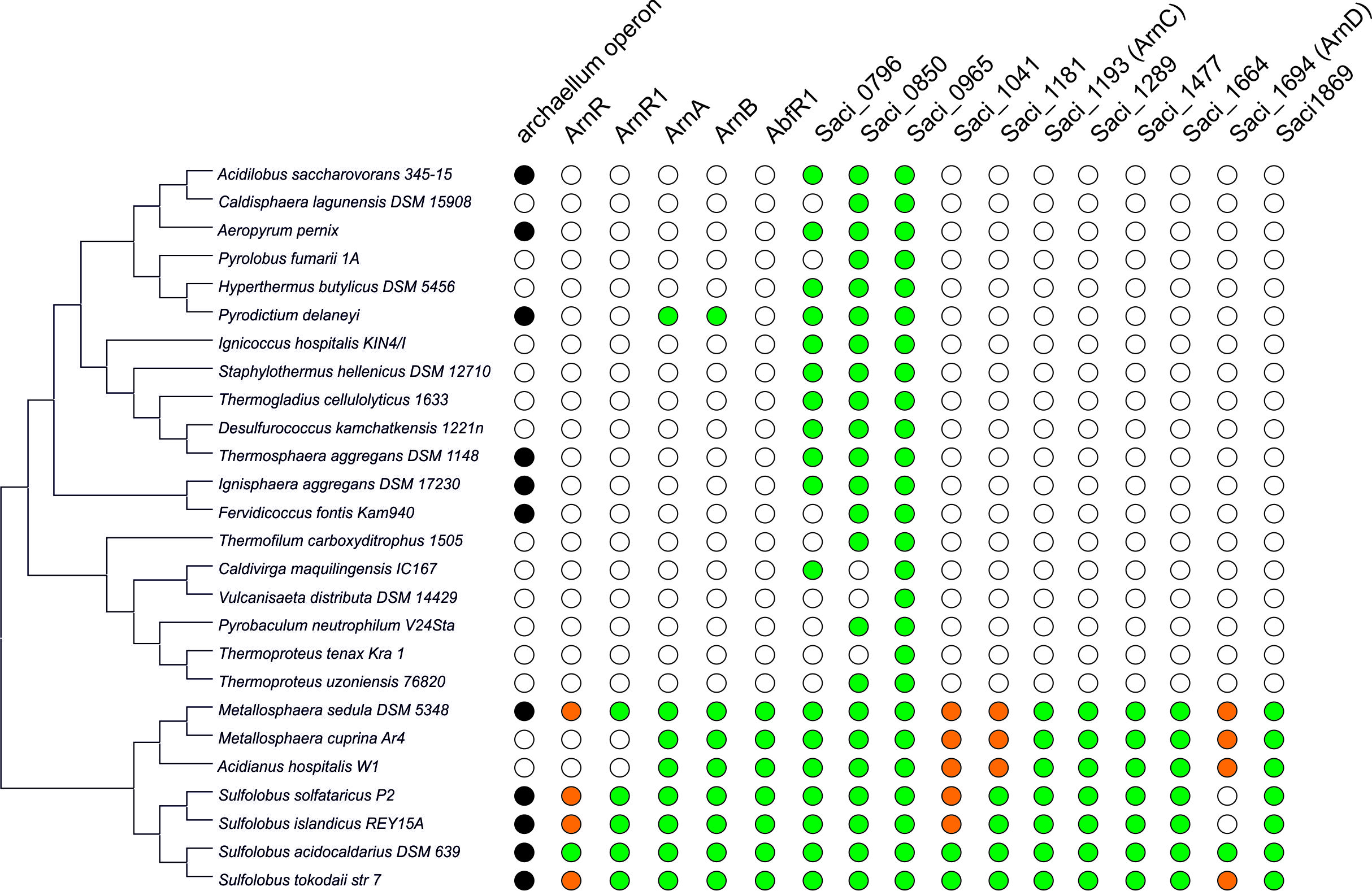
**

**Fig. S2. Conservation of proteins of the archaellum regulatory network in different crenarchaeal genomes.** To construct a phylogenetic tree of different crenarchaea, 26 fully sequenced crenarchaeael genomes, that were also available via SyntTax (Oberto, 2013)**,** were selected, and the following 16S rRNA sequences were obtained: *Acidilobus saccharovorans* 345-15 (CP001742; nt:176279-177775), *Caldisphaera lagunensis* DSM 15908 (CP003378; nt:664985-666372), *Aeropyrum pernix* (AB263906; nt:1-1388), *Desulfurococcus kamchatkensis* 1221n (CP001140; nt:961526-963021), *Ignicoccus hospitalis* KIN4/I (CP000816; nt:728379-729800), *Ignisphaera aggregans* DSM 17230 (CP002098; nt:1279164-1280671), *Staphylothermus hellenicus* DSM 12710 (CP002051; nt:1270431-1271941), *Thermogladius cellulolyticus* 1633 (CP003531; nt:422343-423841), *Thermosphaera aggregans* DSM 11486 (CP001939; nt:52824-54320), Hyperthermus butylicus DSM 5456 (CP000493; nt:1664038-1665353), *Pyrodictium sp* Su06 (KC145154; nt:1-1290), *Pyrolobus fumarii* 1A (CP002838; nt:86246-84671), *Fervidicoccus fontis* Kam940 (CP003423; nt:1068522-1070023), *Acidianus hospitalis* W1 (CP002535; nt:1296061-1297559), *Metallosphaera sedula* DSM 5348 (CP000682; nt:1705274-1706771), *Metallosphaera cuprina* Ar4 (CP002656; nt:417677-419106), *Sulfolobus solfataricus* P2 (AE006641; nt:871672-873167), *Sulfolobus islandicus* REY15A (CP002425; nt:1035971-1037465), *Sulfolobus acidocaldarius* DSM 639 (CP000077; nt:1107140-1108619), *Sulfolobus tokodaii* str 7 (BA000023; nt:1186506-1187999), *Thermofilum carboxyditrophus* 1505 (GU187356; nt:1-1375), *Caldivirga maquilingensis* IC167 (CP000852; nt:129290-130825), *Pyrobaculum neutrophilum* V24Sta (CP001014; nt:690421-692612), *Thermoproteus tenax* Kra 1 (FN869859; nt:1699104-1700606), *Thermoproteus uzoniensis* 76820 (CP002590; nt:1330555-1332075), *Vulcanisaeta distributa* DSM 14429 (CP002100; nt:631480-63291). The sequences were aligned using MUSCLE and after alignment truncated 5’ before and 3’ after the *S. acidodaldarius* 5’-GGCGGACGGCUGAGUAA-3’ and 5’-CGGGUCAACAACCCGC-3’ sequences. The evolutionary history was inferred by using the Maximum Likelihood method based on the Tamura-Nei model (Tamura and Nei, 1993). The bootstrap consensus tree inferred from 1000 replicates is taken to represent the evolutionary history of the taxa analyzed (Joseph Felsenstein, 1985). Branches corresponding to partitions reproduced in less than 50% bootstrap replicates are collapsed. Initial tree(s) for the heuristic search were obtained automatically by applying Neighbor-Join and BioNJ algorithms to a matrix of pairwise distances estimated using the Maximum Composite Likelihood (MCL) approach, and then selecting the topology with superior log likelihood value. The analysis involved 26 nucleotide sequences. All positions containing gaps and missing data were eliminated. There were a total of 1226 positions in the final dataset. Evolutionary analyses were conducted, and the phylogenetic tree was created in MEGA6 (Tamura *et al.*, 2013). Reciprocal BLASTP analysis was essentially performed as described previously (Huntley *et al.*, 2011). BLASTP analyses were done with the full length *S. acidocaldarius* proteins again a target genome. For queries with an expect value <E-40, BLASTP analyses were performed where the best hit was blasted against the *S. acidocaldarius genome*. If the result of the second BLAST analysis corresponded to the original protein, the target genome was considered to contain an ortholog.


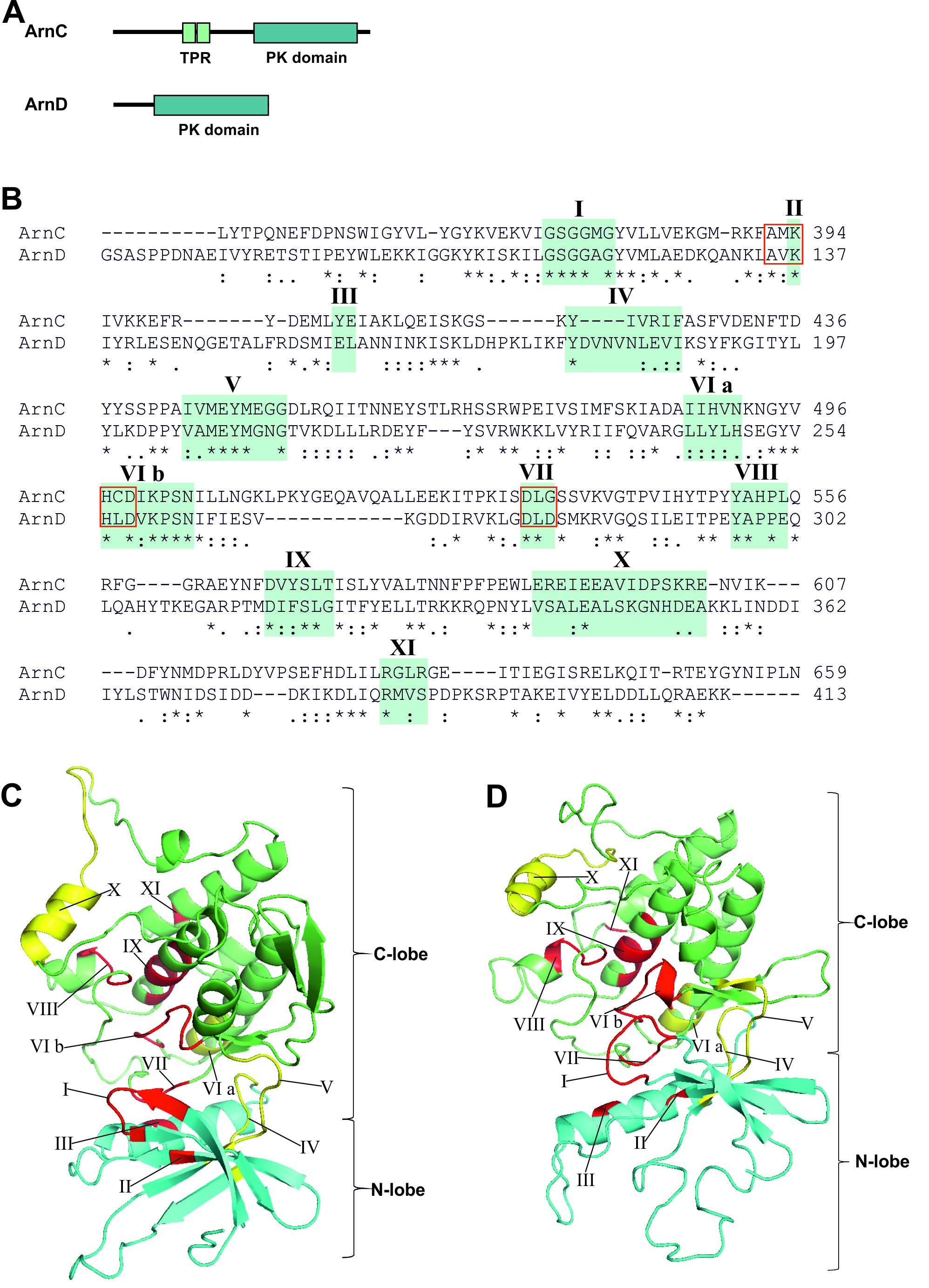


**Fig. S3.** **Conserved domains and modeled structures of ArnC and ArnD.** A. Conserved domains of ArnC and ArnD. TPR (tetratrico peptide repeat), PK (protein kinase domain). B. Sequence alignment of ArnC and ArnD generated with Clustal Omega (Sievers *et al.*, 2014). Highlighted in green and numbered are the twelve subdomains of ePKs. * conserved residues, : strong similarity between residues, . weak similarity between residues. The putative positions of the AXK-, Y/HRD- and DFG-motifs are indicated by red boxes. C. Model of ArnC based on the structure of the dual specificity protein kinase CLK1 (pdb No. : [1z57](file:///W:\lh1033\Kinases\Untitled_Project_2015-05-22\model\01\templates\1z57.1.A.pdb)). D. Model of ArnD based on the serine/threonine-protein kinase TBK1 (pdb No. : [4eut](file:///W:\lh1033\Kinases\Saci1694_2015-05-22\model\02\templates\4eut.1.A.pdb)). In C. and D.: Cyan: N-terminal lobe responsible for nucleotide anchoring and orientation (contains subdomain I- IV), green: C-terminal lobe which is needed for phosphotransfer and substrate binding (contains subdomains V-XI), red: Highly conserved residues that are forming the catalytic core and are involved in ATP binding/orientation as well as ion binding and phsophotransfer, yellow: Subdomains that are not contributing to the catalytic core but rather to structural stability of the kinase domain. Roman numbers highlight the conserved subdomains. Models were generated with Pcons.net (Wallner *et al.*, 2003).


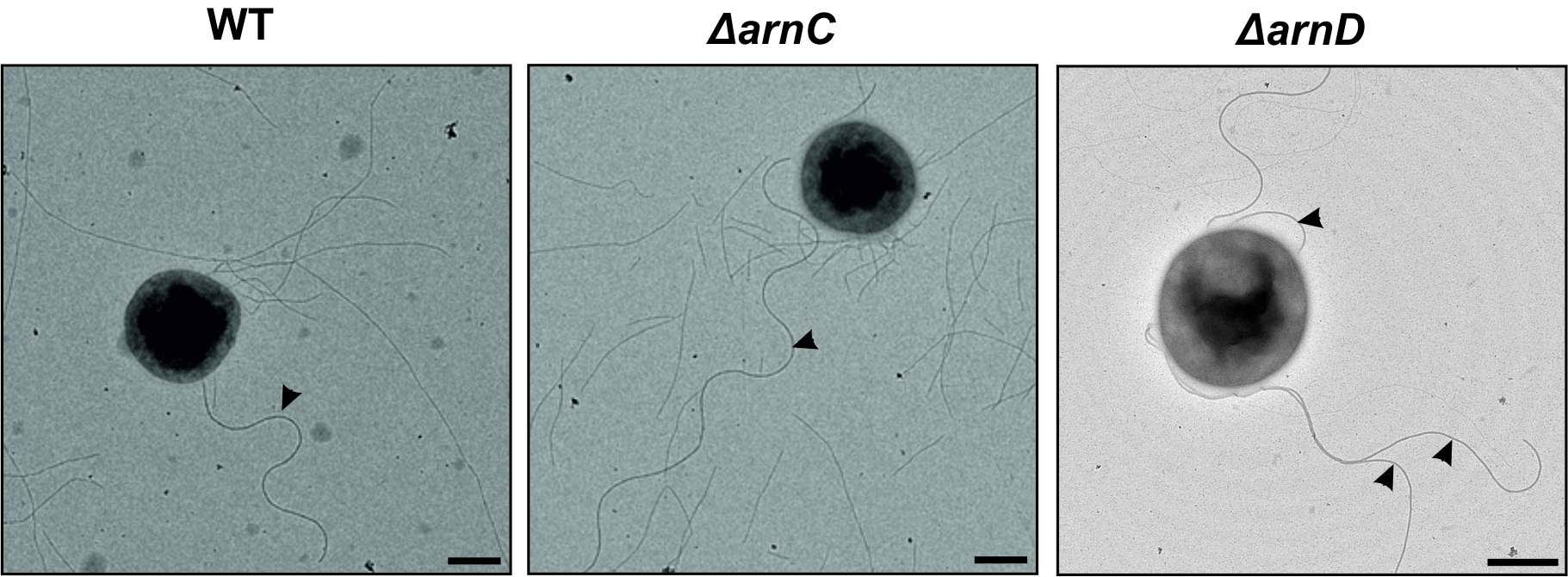


**Fig. S4. Transmission electron microscopy of WT, *ΔarnC* and *ΔarnD* cells after four hours of starvation.** *S. acidocaldarius* strains were starved for four hours as described in material and methods. Cells were harvested at OD_600_ of approximately 0.4, and spin concentrated. Continuous carbon grids (TAAB) were glow-discharged for 60 s at 10 mA. 3 μl cell suspension was deposited on the glow-discharged grids for 60 s, then a suspension of 2% uranyl acetate added for 60 s prior to wicking off and being left to dry. Grids were imaged in a Tecnai G2 Spirit BioTWIN (Tungsten filament) using a 2k x 2k Eagle CCD camera (FEI) and on a Tecnai F20 (FEG filament) at 200 kV equipped with 4k x 4k Falcon II direct electron detector. Black arrows highlight the archaella. All other visible surface structures are archaeal adhesive pili (aap pili). Representative images of each strain are shown. Scale bar = 500 nm.

**Detailed description of experimental procedure and analysis for motility assays**

Strains were grown from -80 C stocks in nutrient rich medium (0.1% NZ-amide and 0.2% dextrin) for two days, diluted ~100 fold and transferred to fresh nutrient rich medium and grown to an OD_600_ between 0.4 and 0.6. Subsequently, cultures were adjusted to an OD_600_ of 0.4 and 5 μl was spotted at room temperature onto semi-solid gelrite plates containing 0.005% of NZ-amide (starvation conditions). After incubation for 10 mins next to the flame, plates were transferred to a humid chamber and incubated at 75°C. After four days plates were scanned on an Epson Perfection scanner (V800 Photo) and images were color inverted and analyzed using CorelDRAW X5.

To quantify the motility, the diameter of the colony and the diameter of the swimming area were determined. Since not all colonies are completely round the diameter of the colony and swimming area are measured horizontally and vertically and subsequently averaged (Fig. S5, black and red lines). The swimming radius was calculated by subtracting the averaged diameter of the colony from the average diameter of the swimming area and dividing this value by two. This procedure was repeated for multiple plates and statistical significance compared to the motility of the MW001 strain (WT) was calculated using a two-tailed student’s t-test with unequal variance.


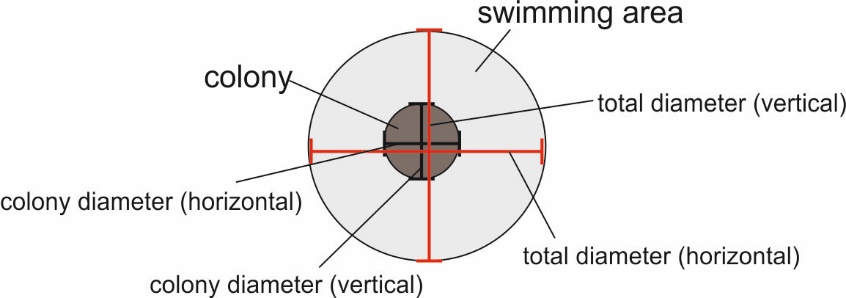

$$swimming radius \left( calculated \right)=\frac{average total diameter-average colony diameter}{2}$$

**Fig. S5** **Calculation of swimming radius for motility assays described above.**


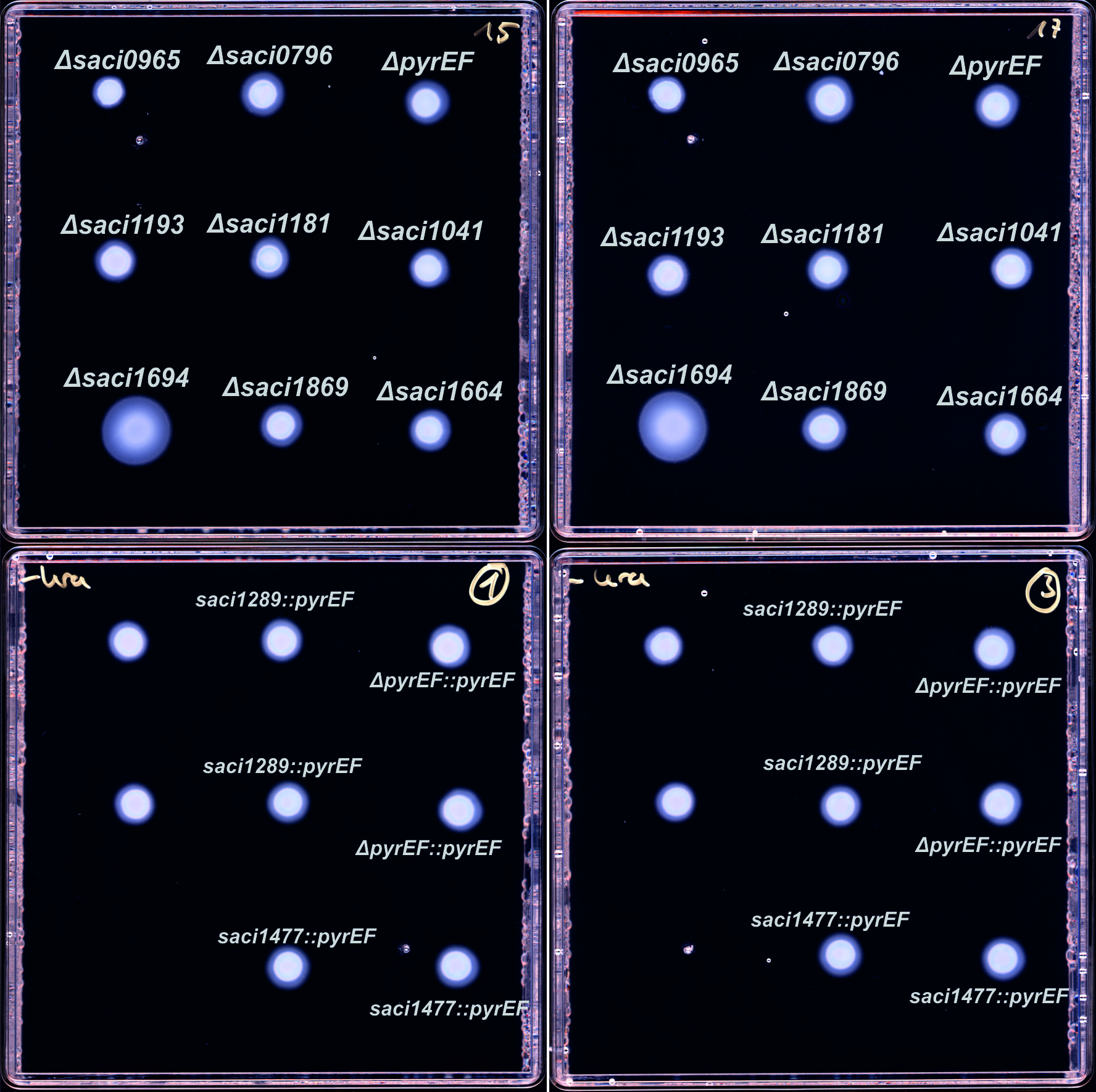


**Fig. S6 Example plates for motility assay.** Plates of the clean deletion mutants (upper lane) and the marker cassette (*pyrEF*) insertion mutants (lower lane) with the respective background strain are shown. Unlabeled colonies are not related to the experiment shown in Fig. 1.

**Table S1:** **Strains**

| Strain | Genotype | Reference |
| --- | --- | --- |
| *E. coli* Top10 | F- mcrA Δ(mrr-hsdRMS-mcrBC) φ80lacZΔM15 ΔlacX74 nupG recA1 araD139 Δ(ara-leu)7697 galE15 galK16 rpsL(Str^R^) endA1 λ^-^ | Invitrogen |
| *E. coli* BL21 (DE3) RIL | B F- *ompT* hsdS(rB- mB-) *dcm+* Tetr *E. coli* gal λ (DE3) *endA* Hte [*argU ileY leuW* Cam^R^] | Stratagene |
| *E. coli* Rosetta (DE3) pLysS | F^-^ *ompT* hsdS_B_(R_B_^-^ m_B_^-^) *gal dcm* λ(DE3 [lacI lacUV5-T7 gene 1 ind1 sam7 nin5]) pLysSRARE (Cam^R^) | Merck |
| *E. coli* ER1821 | F^-^ glnV44 e14^-^(McrA^-^) rfbD1? relA1? endA1 spoT1? thi-1 Δ(mcrC-mrr)114::IS10 | New England Biolabs |
| MW001 | *S. acidocaldarius* DSM639 *ΔpyrEF* | (Wagner *et al.*, 2009) |
| MW268 | Chromosomal conplementation of *pyrEF* deletion in MW001 | (Orell *et al.*, 2013) |
| MW156 | MW001 *ΔaapF* (*saci2318*) | (Henche *et al.*, 2012) |
| MW332 | MW001 *ΔarnR* (*saci1180*), *ΔarnR1* (*saci1171*) | (Lassak *et al.*, 2013) |
| MW012 | MW001 *ΔarnS* (*saci1181*) | Haurat *et al*., unpublished |
| MW382 | MW001 *saci1289::pyrEF* | This study |
| MW384 | *MW001 saci1477::pyrEF* | This study |
| MW368 | MW001 *Δsaci0796* | This study |
| MW394 | MW001 *Δsaci0965* | This study |
| MW360 | MW001 *Δsaci1041* | This study |
| MW354 | MW001 *ΔarnC* (*saci1193*) | This study |
| MW366 | MW001 *Δsaci1664* | This study |
| MW391 | MW001 *ΔarnD* (*saci1694*) | This study |
| MW358 | MW001 *Δsaci1869* | This study |
| MW395 | MW001 *ΔarnC* (*saci1193*), *ΔarnD* (*saci1694*) | This study |

**Table S2:** **Primer**

| Primer | 5’-3’ sequence | Purpose/Target |
| --- | --- | --- |
| RT PCR Primer | | |
| 696 | CTCTAATTTTAACGTCTCAGTAACTAGC | *aapA* (*saci2314*) fw |
| 697 | CCTACTTGTTCCATAGGATTGTTAGG | *aapA* (*saci2314*) rev |
| 1424 | ACTGCGTCTACTGCGTTATCTTTATC | *flaB* (*saci1179*) fw |
| 1425 | GGAGATAAGTCTACACTAGATACACCAGAA | *flaB* (*saci1179*) rev |
| 1426 | GCAGTTGAAGAGTTAGCCTTATCTGTG | *flaX* (*saci1178*) fw |
| 1427 | CCTACTAACTGACTTACGGTACTAATCT | *flaX* (*saci1178*) rev |
| 1480 | CCTGCAACATCTATCCATAACATACCGA | *secY* (*saci0574*) fw |
| 1481 | CCTCATAGTGTATATGCTTTAGTAGTAG | *secY* (*saci0574*) rev |
| 2488 | AGTAGCCTATGGTCTTTCTGAATC | *abfR1* (*saci0446*) fw |
| 2489 | TCAACTAATCCTGCATCTGAAAGC | *abfR1* (*saci0446*) rev |
| 3464 | GCTTTATCTAGACTCAATCCATCGTGA | *arnR* (*saci1180*) fw |
| 3465 | CGGTCTTAAACATAGAAGTGATAAACGG | *arnR* (*saci1180*) rev |
| 3512 | CTCCTGACTACCAACTGACTATTTATC | *aapF* (*saci2318*) fw |
| 3513 | GTTCACCAGTAGAATAGCTCTTTACAC | *aapF* (*saci2318*) rev |
| 3290 | GAACCACAAGCACAAGGAGTAG | *arnA* (*saci1210*) fw |
| 3291 | GGTTGAGGTTGAGCTGGTTCTG | *arnA* (*saci1210*) rev |
| 3292 | AGCCTACTGACGTACCTGAGAC | *arnB* (*saci1211*) fw |
| 3293 | TCCTATTTCCGTAACCGCTGAC | *arnB* (*saci1211*) rev |
| 4711 | CTGCAAAGATTCGGTGGAAG | *arnC* (*saci1193*) fw |
| 4712 | TTCGTCAACGCAACGTAAAG | *arnC* (*saci1193*) rev |
| 4716 | TTTATAGGGAGACCAGCACAATC | *arnD* (*saci1694*) fw |
| 4717 | CATAGCCTGCTCCACCACTAC | *arnD* (*saci1694*) rev |
| Primer for construction of saci1289::pyrEF fragment | | |
| 3238 | CATAAATTTCTAAGGAAGAGACCTACATGGATGCAGACATGAAAAGTATTGACTTTGAGCAGTTCTAG | *saci1289::pryrEF* fw |
| 3239 | CATTATGAAAAAGAAGATCACTATAACAATAGAATGATTTTTTCTAAAGGGACCGGCTATTTTTTCAC | *saci1289::pryrEF* rev |
| Primer for construction of saci1477::pyrEF fragment | | |
| 3242 | GTAGTGGATAGTGGAGATAGGAAAATAGTACTAAAATGTTACAATTCCTCTTTGAGCAGTTCTAG | *saci1477::pryrEF* fw |
| 3243 | GTGGAATAAGGCTAATTAGTGAAATATATTTTATATCGAATATCTCCTTCGGACCGGCTATTTTTTCAC | *saci1477::pryrEF* rev |
| Primer for construction of pSVA1088 | | |
| 1671 | GGTCCATGGGATAGACGAAAGGAAAGAAGAG | *saci0796* upstream fw, *NcoI* site |
| 1672 | CCTAAGTTAGATCTCGAGAAGCCTATAATCCATAGG | *saci0796* upstream rev |
| 1673 | GGATTATAGGCTTCTCGAGATCTAACTTAGGGATATAACC | *saci0796* downstream fw |
| 1674 | GGTCTGCAGCTCGGAGCATATACTATAAATAC | *saci0796* downstream rev, *PstI* site |
| Primer for construction of pSVA2287 | | |
| 4743 | GATCGGCCGGGTAACTCGTCACAAATAAGT | *saci0965* upstream fw, *EagI* site |
| 4735 | ATTAATATTTCGAATTCGTGACAAAGAATTATCCTC | *saci0965* upstream rev |
| 4736 | AATTCTTTGTCACGAATTCGAAATATTAATTCTTTC | *saci0965* downstream fw |
| 4737 | GATGGATCCTTGTTTAATACGGAAGAGGTC | *saci0965* downstream rev, *BamHI* site |
| Primer for construction of pSVA1019 | | |
| 1527 | GGGGGGATCCATACACTTAAGGCTGATGTC | *saci1041* upstream fw, *BamHI* site |
| 1528 | GTTAAGTTAGCTCATGAAAGCCTGTTCATTTCTGATCATC | *saci1041* upstream rev |
| 1529 | GATGATCAGAAATGAACAGGCTTTCATGAGCTAACTTAAC | *saci1041* downstream fw |
| 1530 | GGGGCTGCAGTTCGAAGATGAAGTGTATAAAG | *saci1041* downstream rev, *PstI* site |
| Primer for construction of pSVA1038 | | |
| 1519 | GGGGGGCGGCCGCTACATCTTGCTCGTTGAAA | *saci1193* upstream fw, *NotI* site |
| 1520 | GAGAAGTCAGATTAGATCTCTAAATCCATCCTAATTGTTGG | *saci1193* upstream rev |
| 1521 | CCAACAATTAGGATGGATTTAGAGATCTAATCTGACTTCTC | *saci1193* downstream fw |
| 1522 | GGGGGCTGCAGGGACCGTTACACGATAATAG | *saci1193* downstream rev, *PstI* site |
| Primer for construction of pSVA1091 | | |
| 1687 | GGACCATGGATAGCGGCAATACTGGAGAC | *saci1664* upstream fw, *NcoI* site |
| 1688 | CTAAAGGGCTATGACGTCCACTTGCTCATTATTTAG | *saci1664* upstream rev |
| 1689 | AATGAGCAAGTGGACGTCATAGCCCTTTAGACATATCTC | *saci1664* downstream fw |
| 1690 | GGAGGATCCCTAGCTGTCTGGTGCATAC | *saci1664* downstream rev, *BamHI* site |
| Primer for construction of pSVA1080 | | |
| 1656 | GAACCATGGAGCGGGTCTGGTGGGAATTG | *saci1694* upstream fw, *NcoI* site |
| 1657 | TCTTTGTAATAAGTCATCAAGAACCTTCCTAGAAGACCTTGGCGC | *saci1694* upstream rev |
| 1658 | AGGTCTTCTAGGAAGGTTCTTGATGACTTATTACAAAGAGCAGAG | *saci1694* downstream fw |
| 1659 | GGACTGCAGCAAGCTTATCCTCAATATACCC | *saci1694* downstream rev, *PstI* site |
| Primer for construction of pSVA1087 | | |
| 1679 | GGTCCATGGTGCATAAGGTCTATCCTTTC | *saci1869* upstream fw, *NcoI* site |
| 1680 | CTCACTTCTCCAGCACGTCTCCCATTAGTTAGAGATG | *saci1869* upstream rev |
| 1681 | CTAACTAATGGGAGACGTGCTGGAGAAGTGAGAAGTG | *saci1869* downstream fw |
| 1682 | GGTGGATCCAGGAGGGACAGCTTTGTAAC | *saci1869* downstream rev, *BamHI* site |
| Primer for construction of pSVA3203 | | |
| 4782 | GCGCCGCGGGATTTGCTCGAAAGGTTAAG | *saci1193* with own promoter fw, *SacII* site |
| 4788 | GGCGGGCCCTCAAGCGTAATCTGGAACATCGTATGGGTAGGAACCGATCTCTGAGTAGTTAAGAGG | *saci1193* with own promoter rev, *ApaI* site, HA-tag |
| Primer for construction of pSVA3208 | | |
| 4798 | GCGCCGCGGAGAAGATCTCTGCATTTAGG | *saci1694* with own promoter fw, *SacII* site |
| 4799 | GGCGGGCCCTCAAGCGTAATCTGGAACATCGTATGGGTAGGAACCTTTCTTCTCTGCTCTTTGTA | *saci1694* with own promoter rev, *ApaI* site, HA-tag |

**Table S3: Plasmids**

| Plasmid | Genotype | Reference |
| --- | --- | --- |
| pSA4 | Derived from pET15b, contains the multiple cloning site and N-terminal hexa-His tag of pSA5 | (Albers *et al.*, 2003) |
| pETDuet-1 | *E. coli* expression plasmid containing replicon CloE1 (pBR322) and two MCS, Amp^r^ Car^r^ | Novagene |
| pSVA406 | pGEM-T easy based gene targeting plasmid for construction of S. acidocaldarius deletion mutants, *pyrEF_SSO_* cassette | (Wagner et al. 2012) |
| pSVA1551 | *S. acidocaldarius* expression plasmid, maltose inducible promoter, C-terminal StrepII and His10 tag, Amp^r^ | Wagner A. *et al*, unpublished |
| pSVA1561 | *S. acidocaldarius* expression plasmid, maltose inducible promoter, C-terminal HA-tag, contains *lacS*, Amp^r^ | Wagner A. *et al*, unpublished |
| pSVA1009 | *arnC* (*saci1193*) with N-terminal His-tag cloned into MCSI of pETDuet-1 with *BclI*/*BamHI*, *PstI* | (Reimann *et al.*, 2012) |
| pSVA1019 | In-frame deletion of *saci1041* cloned into pSVA406 with *BamHI*, *PstI* | This study |
| pSVA1076 | *arnD* (*saci1694*) with C-terminal His-tag cloned into MCSI of pETDuet-1 with *NcoI*, *PstI* | (Reimann *et al.*, 2012) |
| pSVA1036 | *arnB* with C-terminal His-tag cloned into MCSI of pETDuet-1 with *NcoI*, *BamHI* | (Reimann *et al.*, 2012) |
| pSVA1038 | In-frame deletion of *arnC* (*saci1193*) cloned into pSVA406 with *NotI*, *PstI* | This study |
| pSVA1080 | In-frame deletion of *arnD* (*saci1694*) cloned into pSVA406 with *NcoI*, *PstI* | This study |
| pSVA1087 | In-frame deltion of *saci1869* cloned into pSVA406 with *NcoI*, *BamHI* | This study |
| pSVA1088 | In-frame deletion of *saci0796* cloned into pSVA406 with *NcoI*, *PstI* | This study |
| pSVA1091 | In-frame deletion of *saci1664* cloned into pSVA406 with *NcoI*, *BamHI* | This study |
| pSVA2287 | In-frame deletion of *saci0965* cloned into pSVA406 with *EagI*, *BamHI* | This study |
| pSVA3203 | *arnC* (*saci1193*) with own promoter and C-terminal HA-tag cloned into pSVA1551 with *SacII*, *ApaI* | This study |
| pSVA3208 | *arnD* (*saci1694*) with own promoter and C-terminal HA-tag cloned into pSVA1551 with *SacII*, *ApaI* | This study |

**References**

Albers, S., Szabó, Z., and Arnold, J.M. (2003) Archaeal Homolog of Bacterial Type IV Prepilin Signal Peptidases with Broad Substrate Specificity Archaeal Homolog of Bacterial Type IV Prepilin Signal Peptidases with Broad Substrate Specificity. **185**: 3918–3925.

Henche, A.-L., Koerdt, A., Ghosh, A., and Albers, S.-V. (2012) Influence of cell surface structures on crenarchaeal biofilm formation using a thermostable green fluorescent protein. *Environ Microbiol* **14**: 779–93.

Huntley, S., Hamann, N., Wegener-feldbru, S., Treuner-lange, A., Kube, M., Reinhardt, R., *et al.* (2011) Comparative Genomic Analysis of Fruiting Body Formation in Myxococcales. **28**: 1083–1097.

Joseph Felsenstein (1985) Confidence Limits on Phylogenies : An Approach Using the Bootstrap. *Evolution (N Y)* **39**: 783–791.

Lassak, K., Peeters, E., Wróbel, S., and Albers, S.-V. (2013) The one-component system ArnR: a membrane-bound activator of the crenarchaeal archaellum. *Mol Microbiol* **88**: 125–39.

Oberto, J. (2013) SyntTax: a web server linking synteny to prokaryotic taxonomy. *BMC Bioinformatics* **14**: 4.

Orell, A., Peeters, E., Vassen, V., Jachlewski, S., Schalles, S., Siebers, B., and Albers, S.-V. (2013) Lrs14 transcriptional regulators influence biofilm formation and cell motility of Crenarchaea. *ISME J* **7**: 1886–98.

Reimann, J., Lassak, K., Khadouma, S., Ettema, T.J.G., Yang, N., Driessen, A.J.M., *et al.* (2012) Regulation of archaella expression by the FHA and von Willebrand domain-containing proteins ArnA and ArnB in Sulfolobus acidocaldarius. *Mol Microbiol* **86**: 24–36.

Sievers, F., Wilm, A., Dineen, D., Gibson, T.J., Karplus, K., Li, W., *et al.* (2014) Fast, scalable generation of high-quality protein multiple sequence alignments using Clustal Omega. *Mol Syst Biol* **7**: 539–539.

Tamura, K., and Nei, M. (1993) Estimation of the Number of Nucleotide Substitutions in the Control Region of Mitochondrial DNA in Humans and. **10**.

Tamura, K., Stecher, G., Peterson, D., Filipski, A., and Kumar, S. (2013) MEGA6 : Molecular Evolutionary Genetics Analysis Version 6 . 0. **30**: 2725–2729.

Wagner, M., Berkner, S., Ajon, M., Driessen, A.J.M., Lipps, G., and Albers, S.-V. (2009) Expanding and understanding the genetic toolbox of the hyperthermophilic genus Sulfolobus. *Biochem Soc Trans* **37**: 97–101.

Wallner, B., Fang, H., and Elofsson, A. (2003) Automatic consensus-based fold recognition using Pcons, ProQ, and Pmodeller. *Proteins* **53 Suppl 6**: 534–41.
