# Supplementary material for: Expanding the archaellum regulatory network – the eukaryotic protein kinases ArnC and ArnD influence motility of Sulfolobus acidocaldarius
Source: Microbiologyopen. 2016 Oct 22;6(1):e00414. doi: 10.1002/mbo3.414 (PMC5300886; doi:10.1002/mbo3.414)

Annotated MS/MS spectra of phosphorylated ArnB peptides.

T280

Raw file	Scan	Method	Score	m/z
ELITE-RSLC011330	5497	ITMS; CID	133.04	593.78

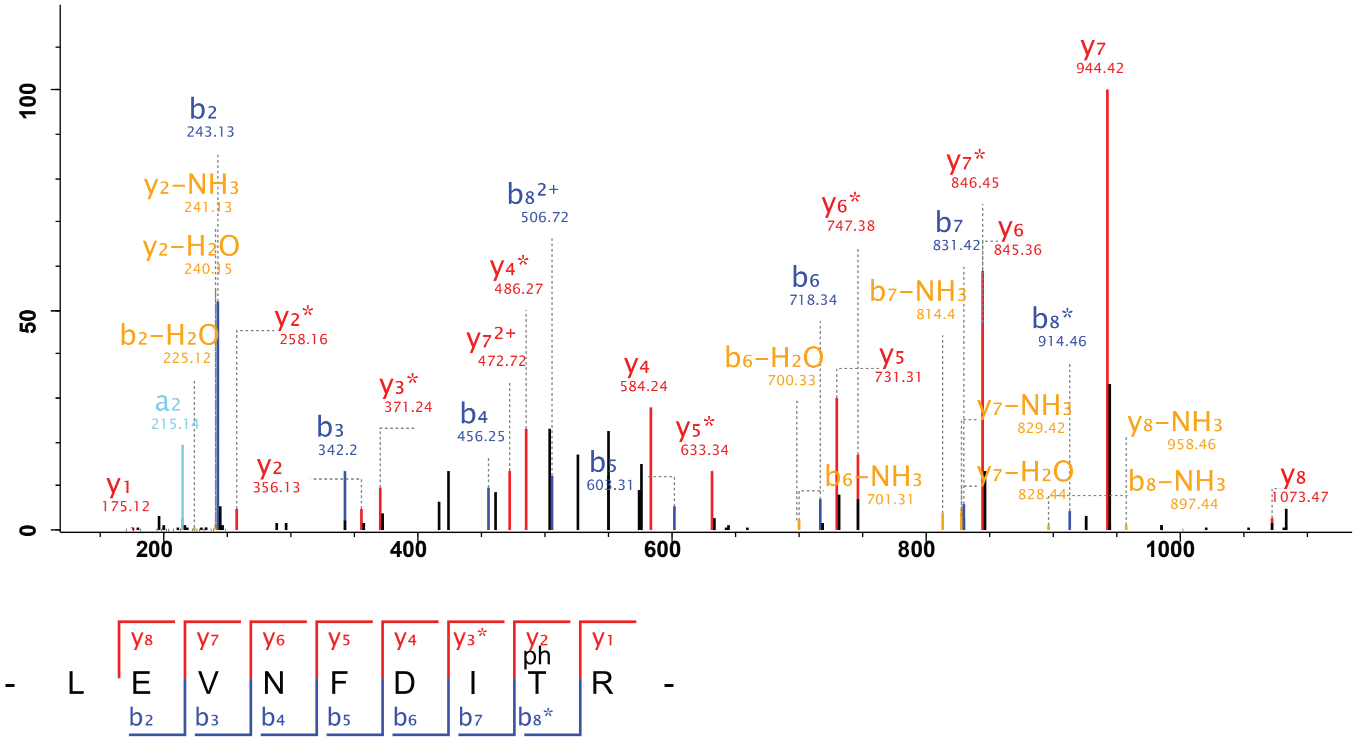

S319

Raw file	Scan	Method	Score	m/z
ELITE-RSLC011329	4490	ITMS; CID	74.12	1175.2

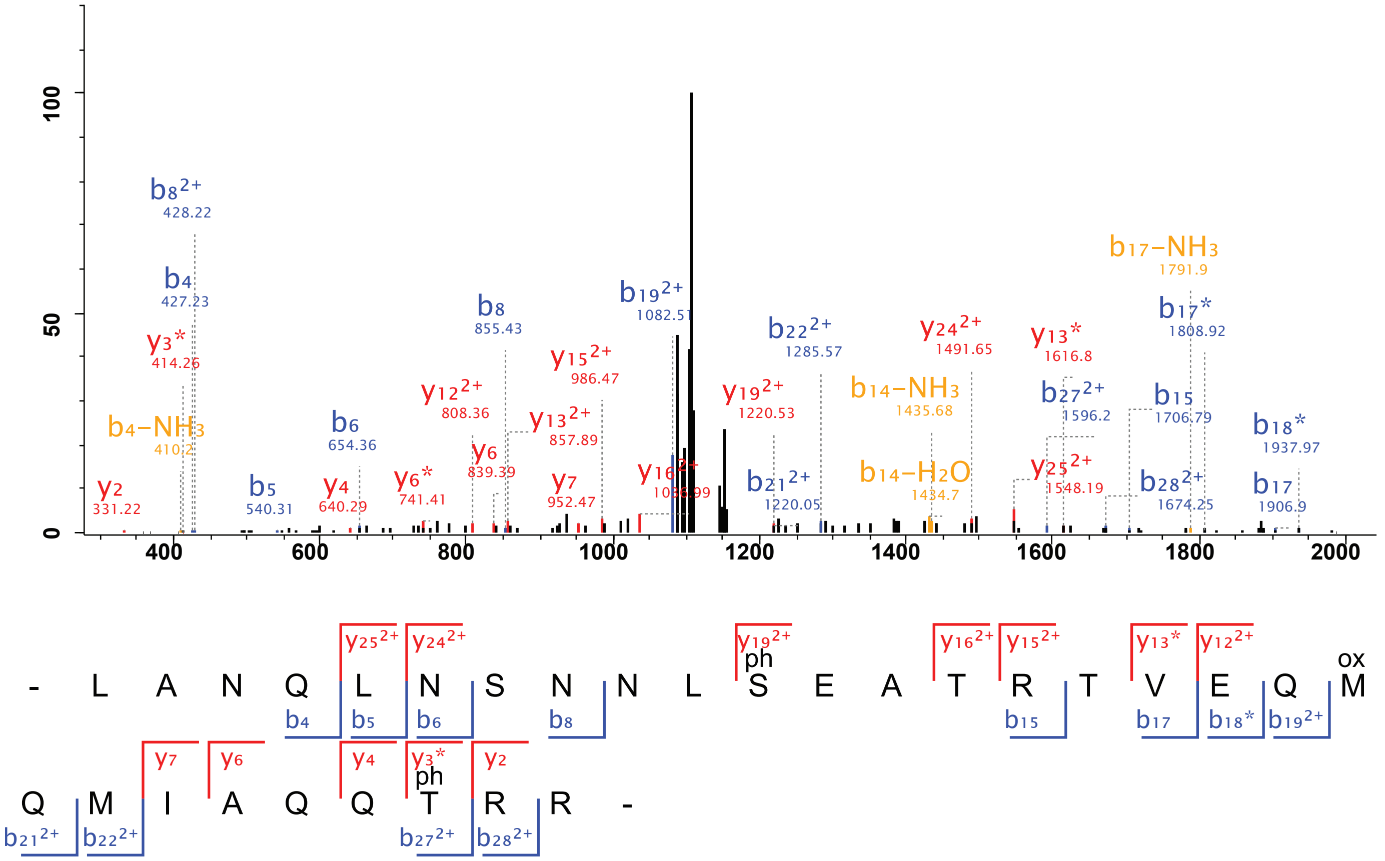

T322

Raw file	Scan	Method	Score	m/z
ELITE-RSLC011324	3817	ITMS; CID	234.62	862.9

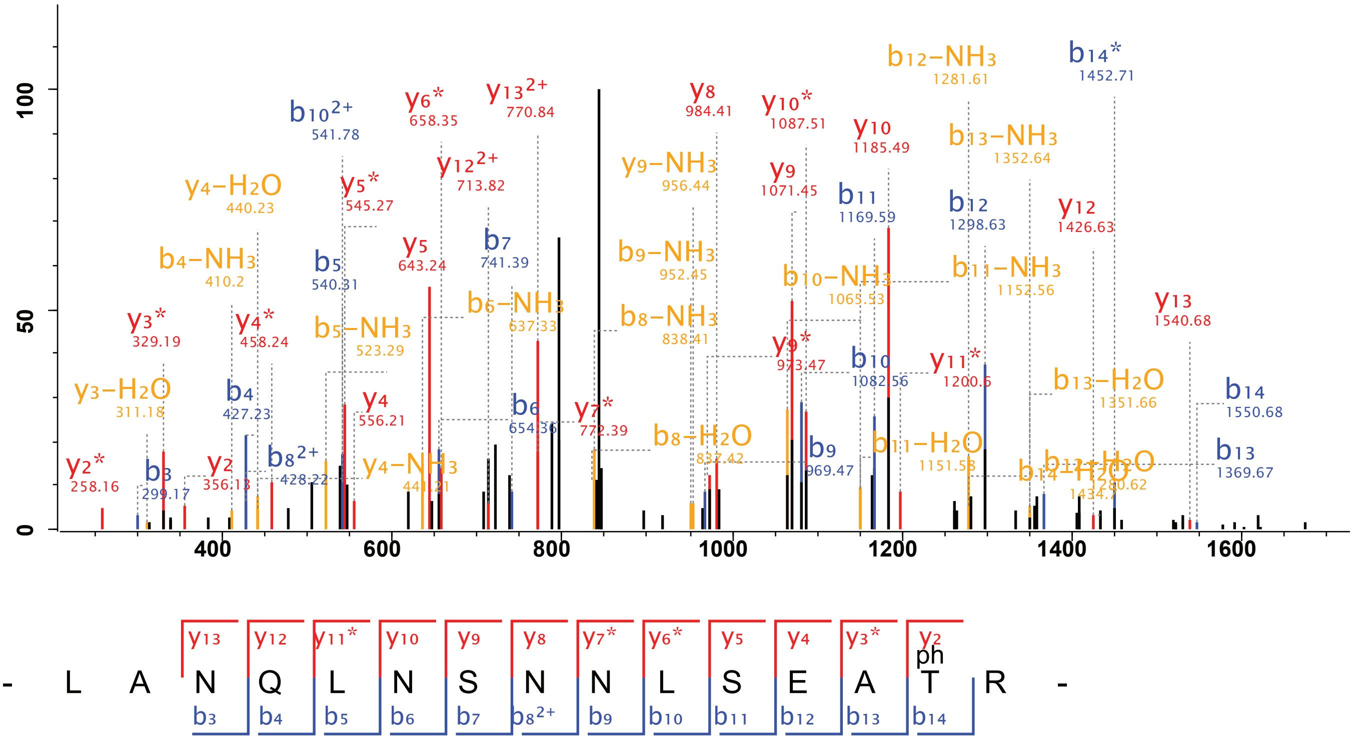

T324

Raw file	Scan	Method	Score	m/z
ELITE-RSLC011323	4119	ITMS; CID	113.69	821.91

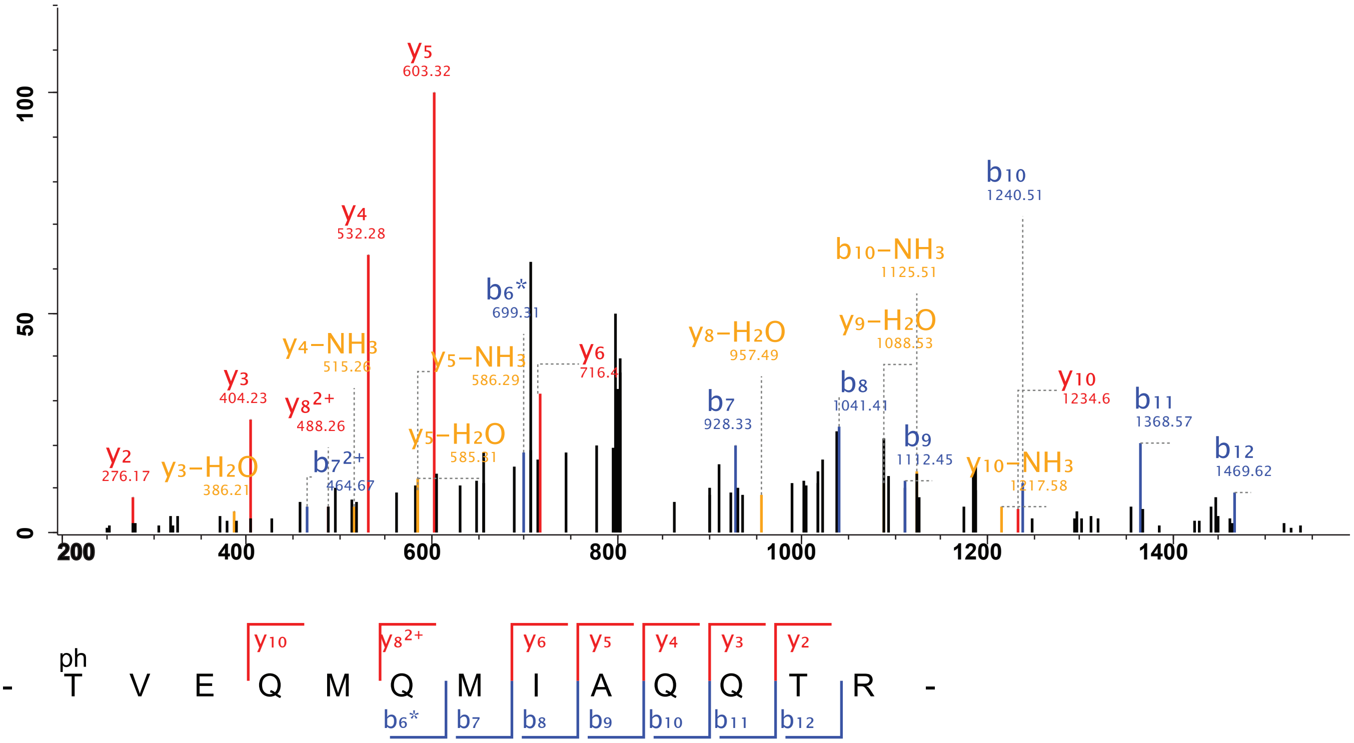

T335

Raw file	Scan	Method	Score	m/z
ELITE-RSLC011329	4408	ITMS; CID	270.95	830.36

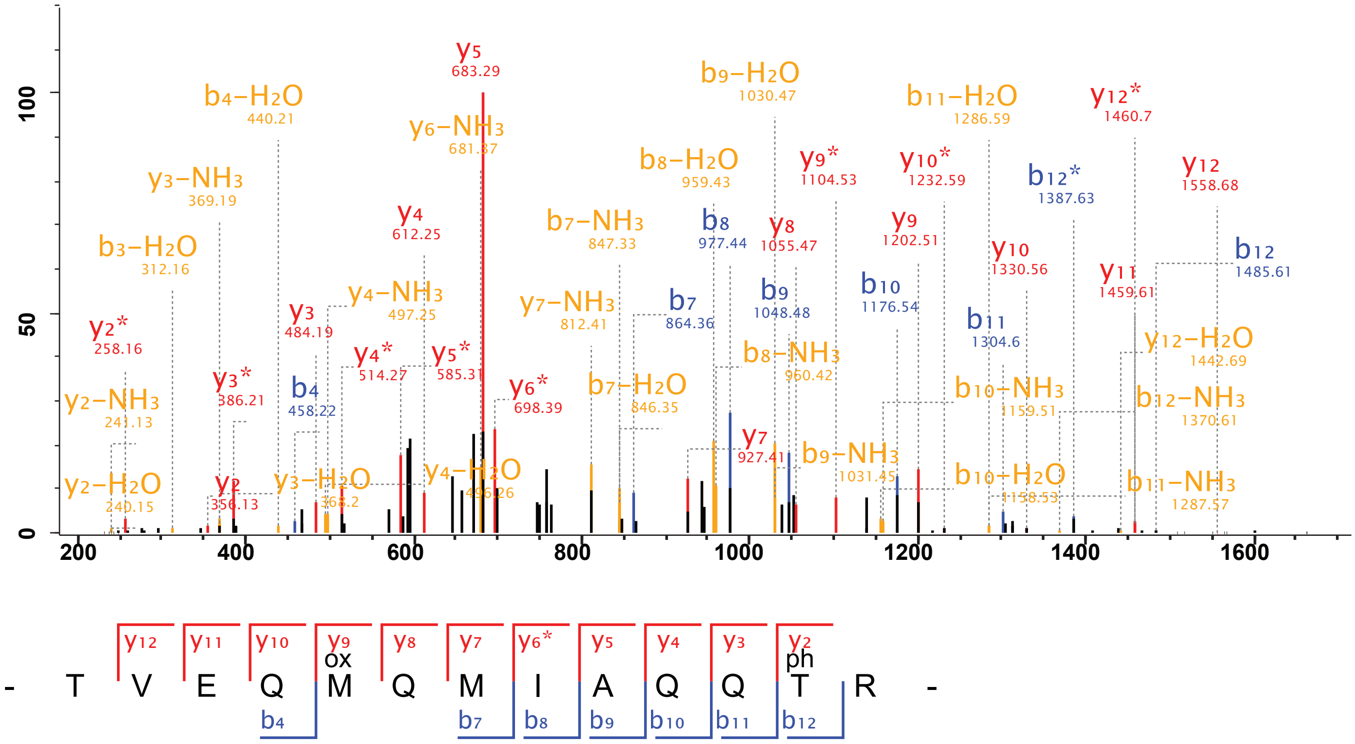

T343

Raw file	Scan	Method	Score	m/z
ELITE-RSLC011330	3634	ITMS; CID	86.11	614.79

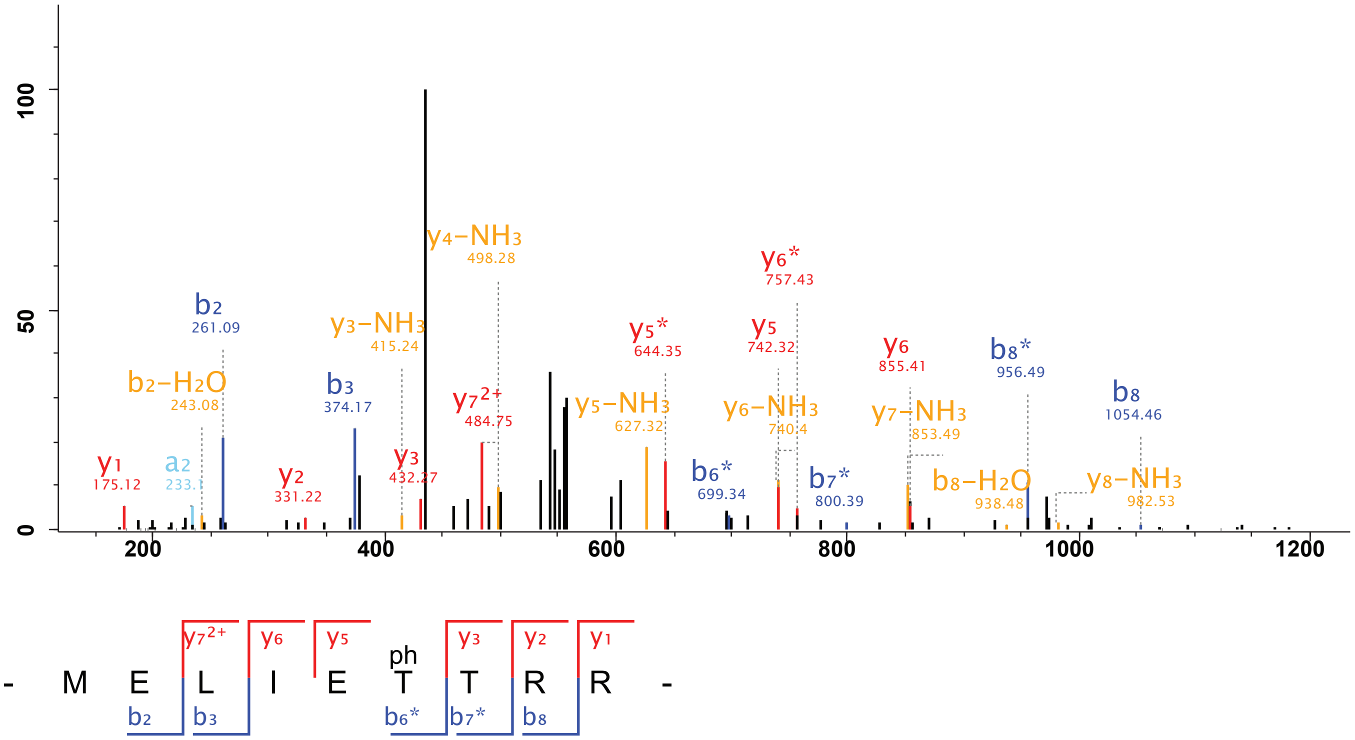

T344

Raw file	Scan	Method	Score	m/z
ELITE-RSLC011328	3656	ITMS; CID	113.95	622.79

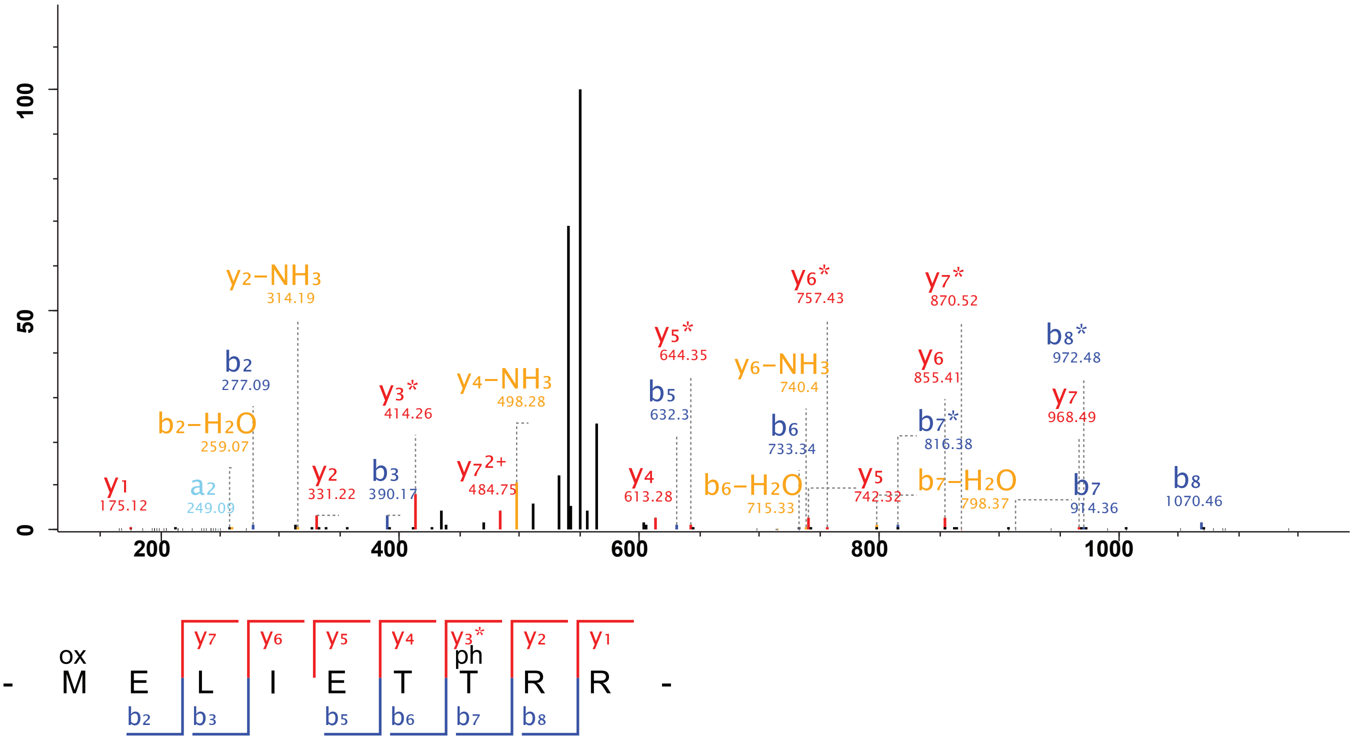

T353

Raw file	Scan	Method	Score	m/z
ELITE-RSLC011330	3452	ITMS; CID	230.81	558.25

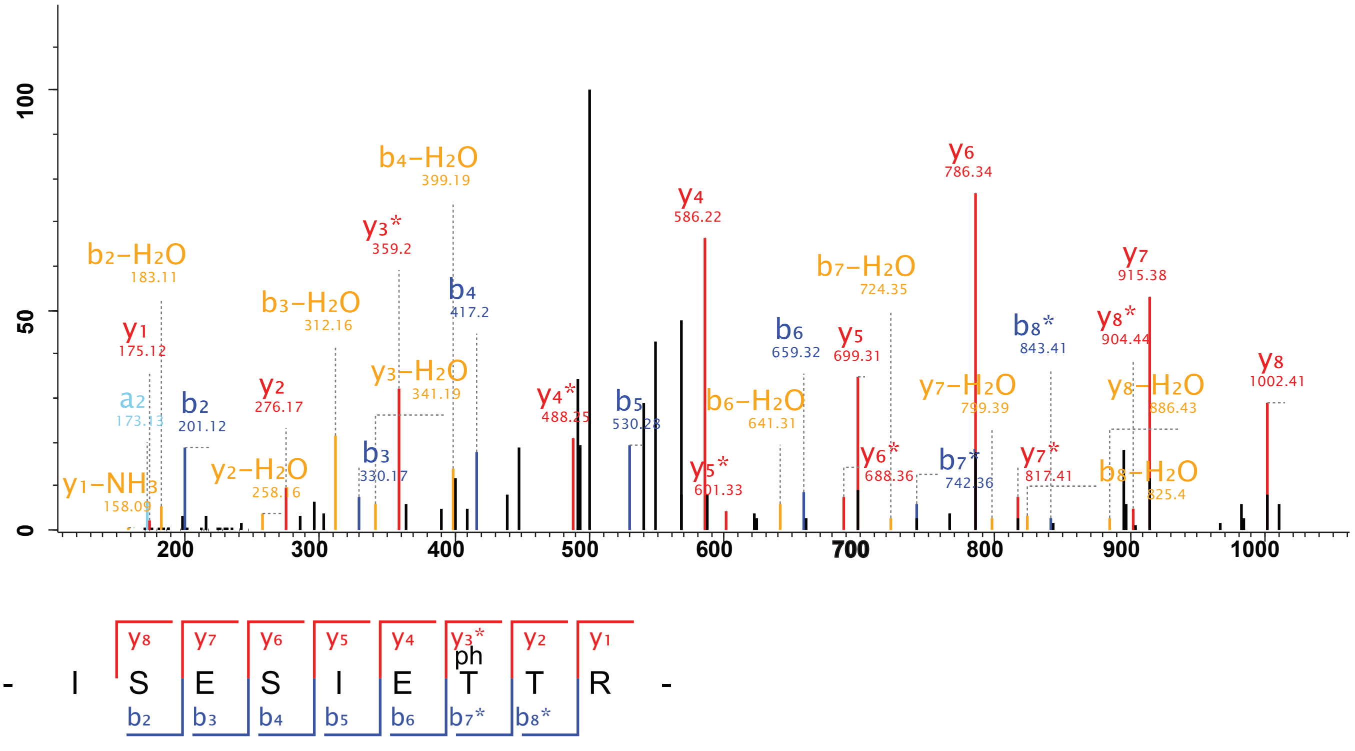

T354

Raw file	Scan	Method	Score	m/z
ELITE-RSLC011323	2810	ITMS; CID	119.62	714.35

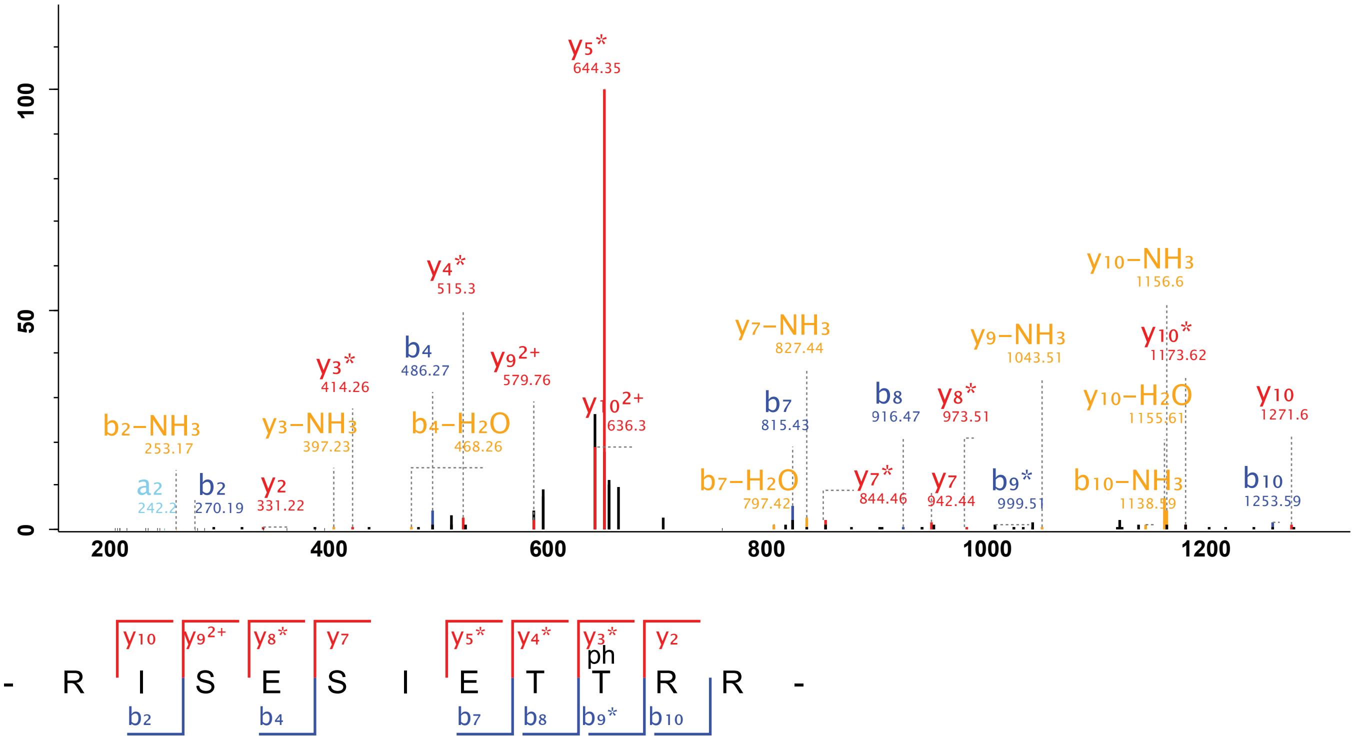

T359

Raw file	Scan	Method	Score	m/z
ELITE-RSLC011329	2946	ITMS; CID	120.29	610.26

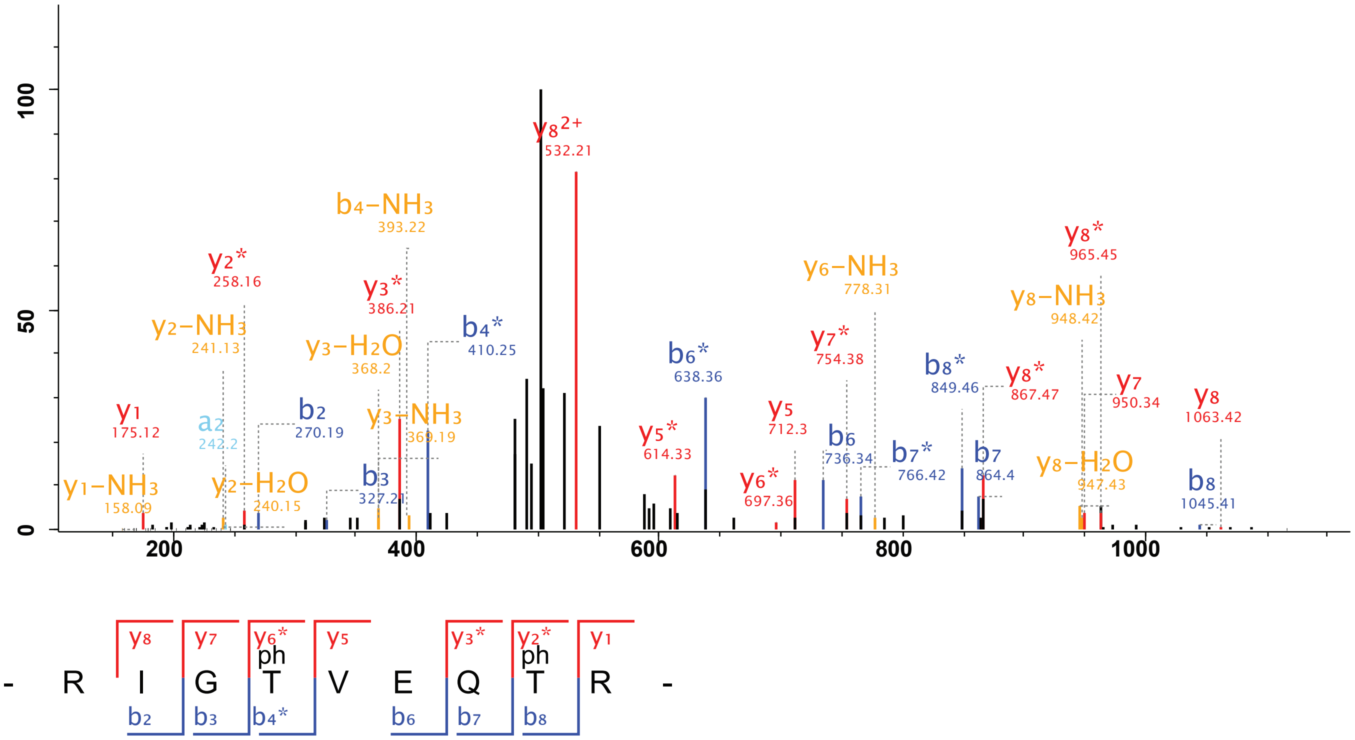

T363

Raw file	Scan	Method	Score	m/z
ELITE-RSLC011328	2877	ITMS; CID	147.58	492.23

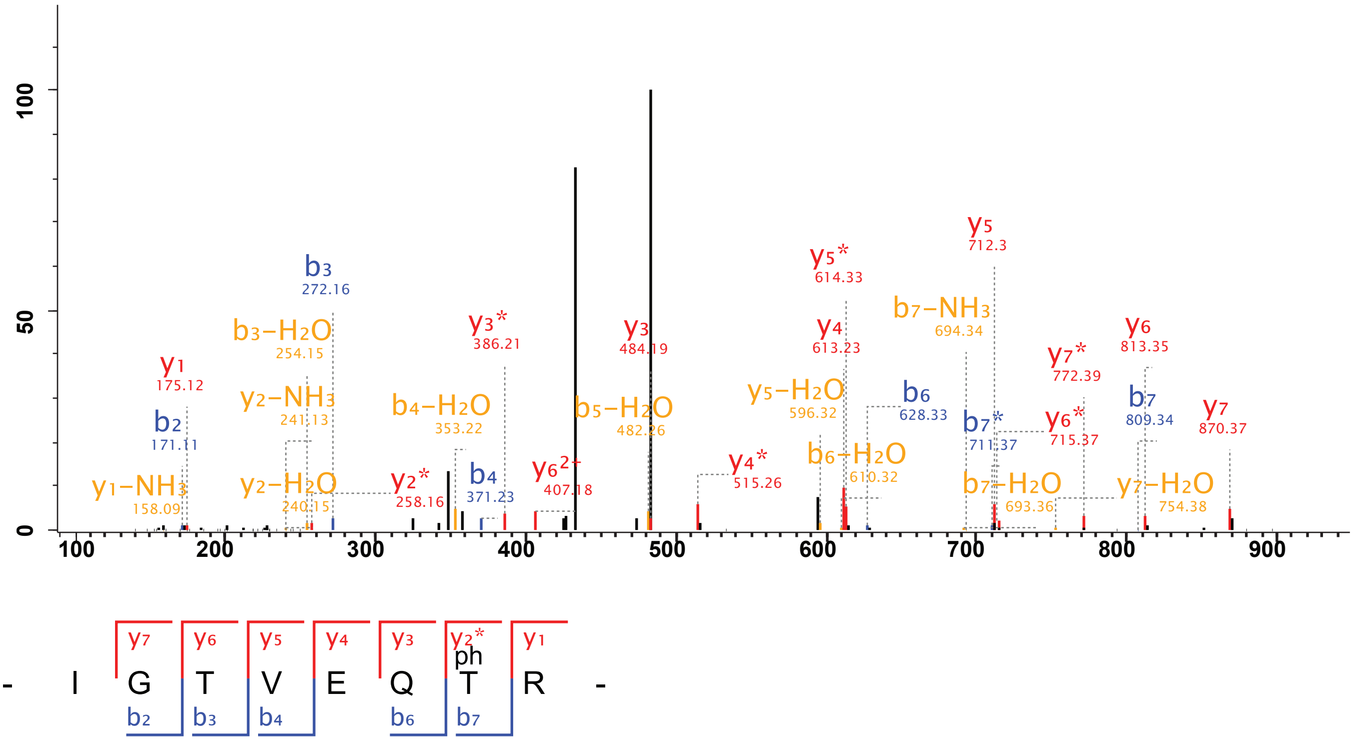

S367

Raw file	Scan	Method	Score	m/z
ELITE-RSLC011329	3281	ITMS; CID	122.97	921.02

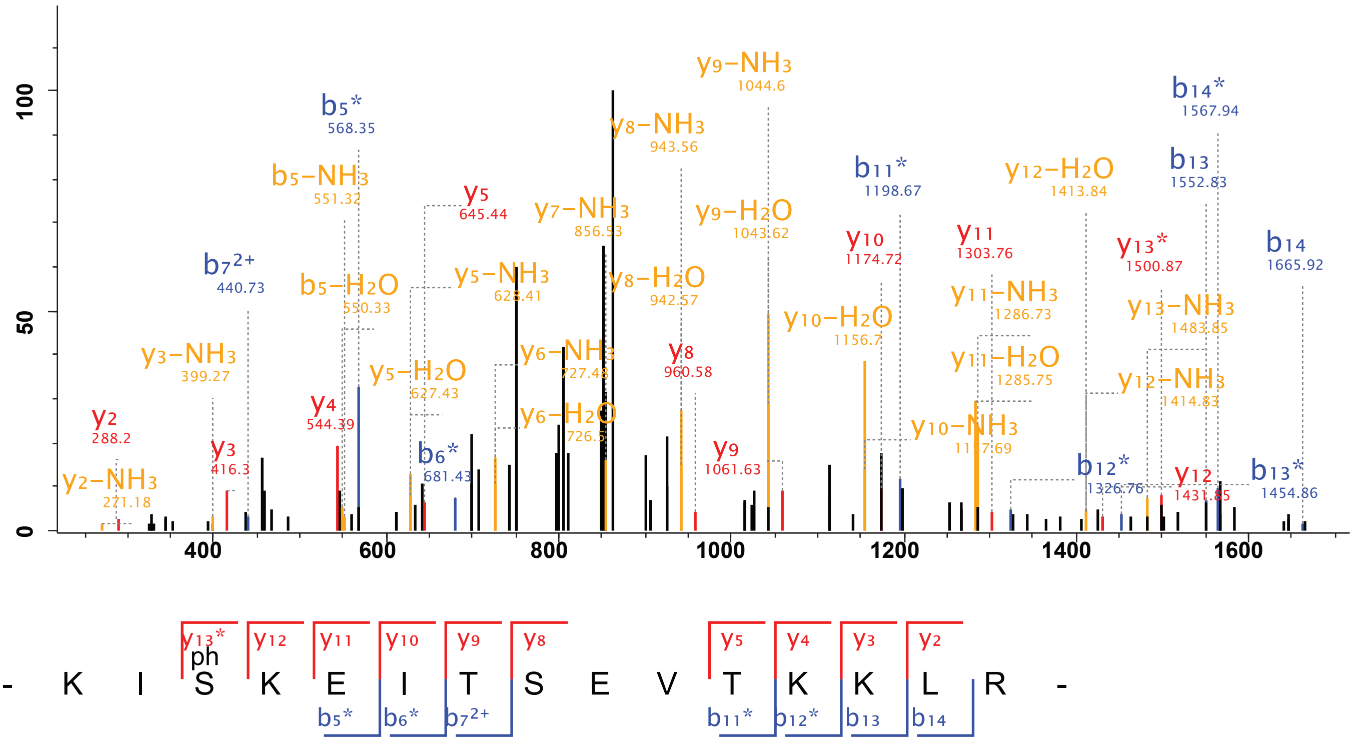

T371

Raw file	Scan	Method	Score	m/z
ELITE-RSLC011329	3352	ITMS; CID	253.42	692.36

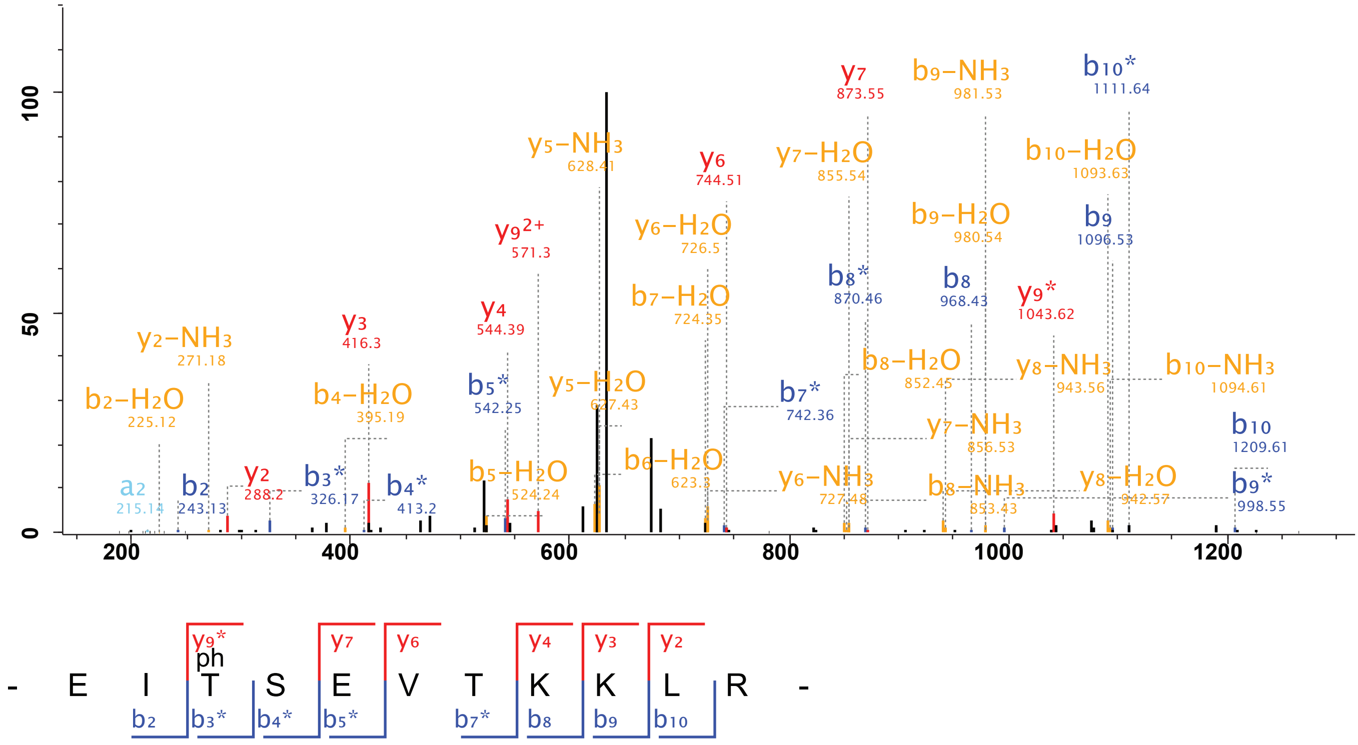

S372

Raw file	Scan	Method	Score	m/z
ELITE-RSLC011325	3444	ITMS; CID	169.09	692.36

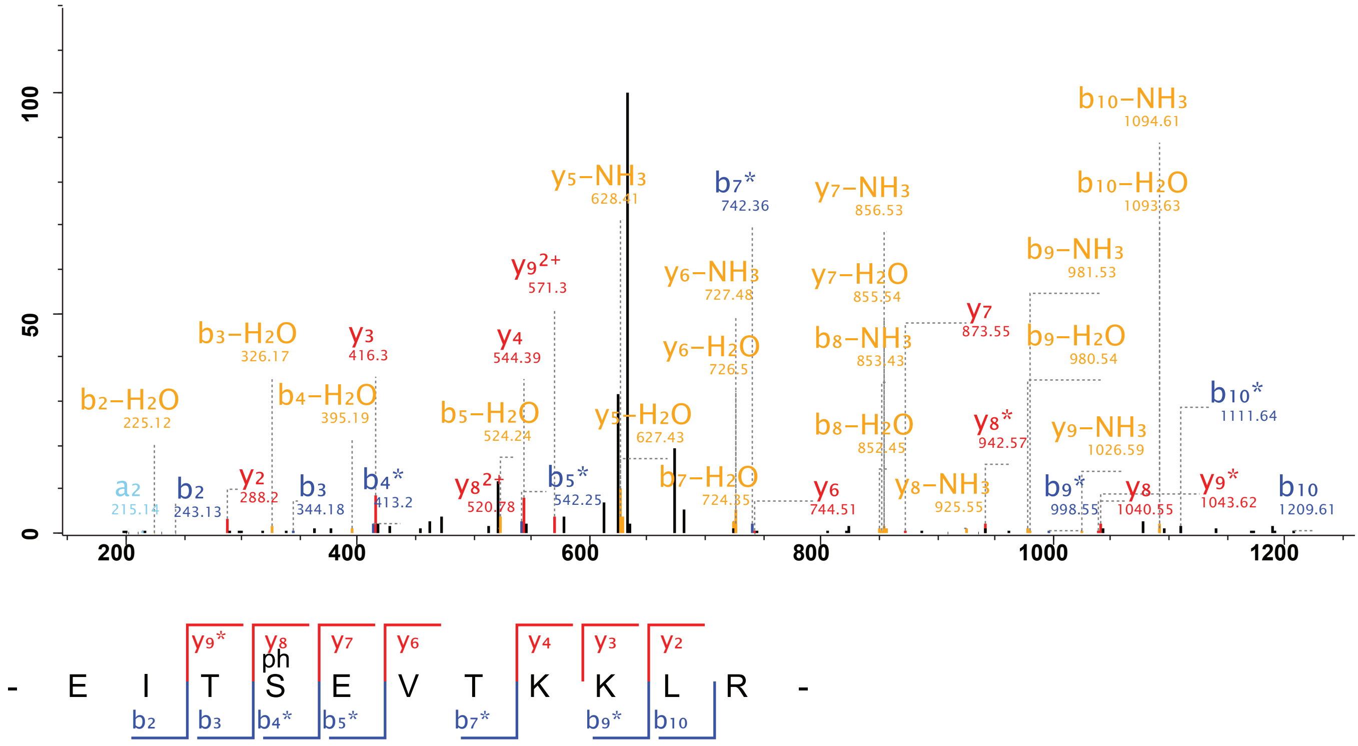

T375

Raw file	Scan	Method	Score	m/z
ELITE-RSLC011325	2963	ITMS; CID	149.31	721.88

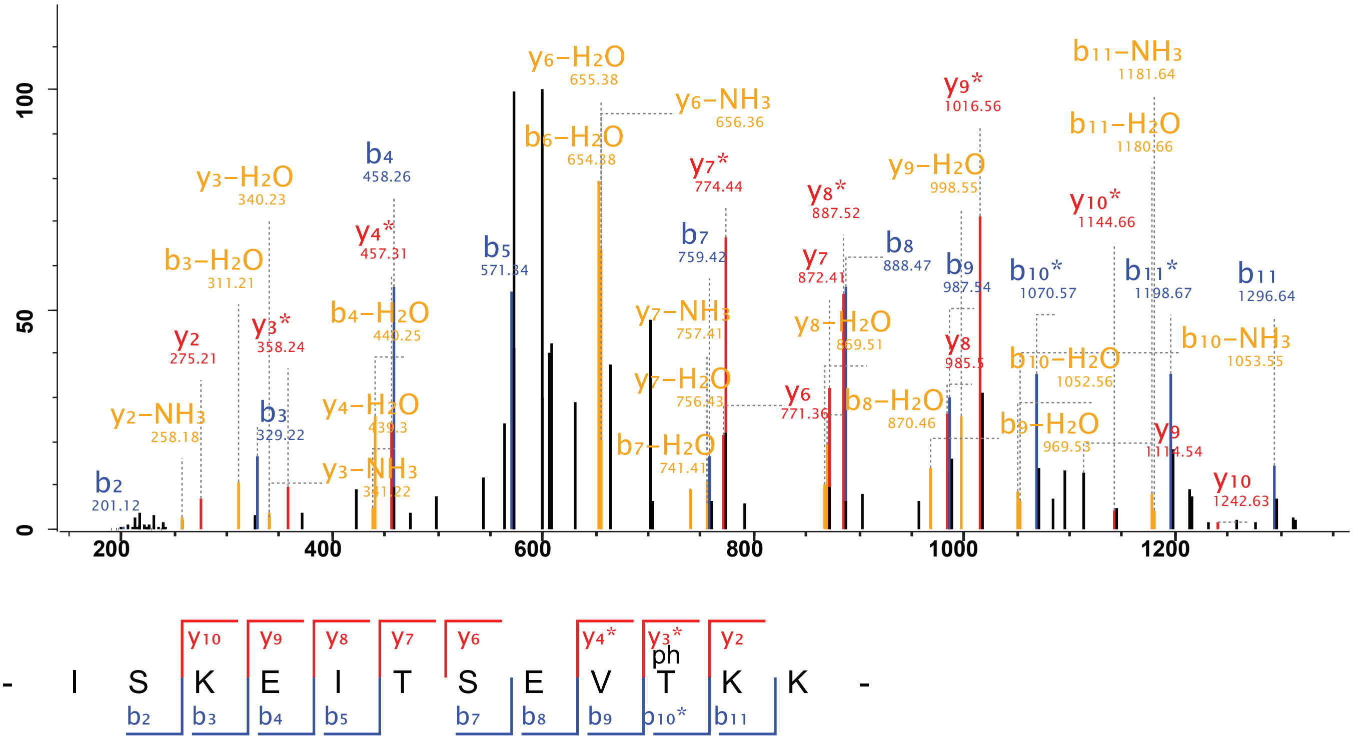

Supplement: Supplementary file 2 [file MBO3-6-0-s002.pdf]
